# The Bactericidal Fatty Acid Mimetic 2CCA-1 Selectively Targets Pneumococcal Extracellular Polyunsaturated Fatty Acid Metabolism

**DOI:** 10.1128/mBio.03027-20

**Published:** 2020-12-15

**Authors:** Elisabeth Reithuber, Priyanka Nannapaneni, Olena Rzhepishevska, Anders E. G. Lindgren, Oleksandr Ilchenko, Staffan Normark, Fredrik Almqvist, Birgitta Henriques-Normark, Peter Mellroth

**Affiliations:** a Department of Microbiology, Tumor and Cell Biology, Karolinska Institutet, Stockholm, Sweden; b Department of Chemistry, Umeå University, Umeå, Sweden; c Umeå Centre for Microbial Research, Umeå University, Umeå, Sweden; d Department of Microbiology, Virology and Biotechnology, Odessa II Mechnikov National University, Odessa, Ukraine; e Department of Clinical Microbiology, Karolinska University Hospital, Stockholm, Sweden; f Singapore Centre for Environmental Life Sciences Engineering SCELSE, Nanyang Technological University, Singapore, Republic of Singapore; g LKC Lee Kong Chian School of Medicine, Nanyang Technological University, Singapore, Republic of Singapore; GSK Vaccines

**Keywords:** *Streptococcus pneumoniae*, pneumococci, LytA, small antimicrobial compound, extracellular fatty acid metabolism, FabT, FakB3, DegV, pneumococcus

## Abstract

Streptococcus pneumoniae, a major cause of pneumonia, sepsis, and meningitis worldwide, has the nasopharynges of small children as its main ecological niche. Depletion of pneumococci from this niche would reduce the disease burden and could be achieved using small molecules with narrow-spectrum antibacterial activity. We identified the alkylated dicyclohexyl carboxylic acid 2CCA-1 as a potent inducer of autolysin-mediated lysis of S. pneumoniae, while having low activity against Staphylococcus aureus. 2CCA-1-resistant strains were found to have inactivating mutations in *fakB3*, known to be required for uptake of host polyunsaturated fatty acids, as well as through inactivation of the transcriptional regulator gene *fabT*, vital for endogenous, *de novo* fatty acid synthesis regulation. Structure activity relationship exploration revealed that, besides the central dicyclohexyl group, the fatty acid-like structural features of 2CCA-1 were essential for its activity. The lysis-inducing activity of 2CCA-1 was considerably more potent than that of free fatty acids and required growing bacteria, suggesting that 2CCA-1 needs to be metabolized to exert its antimicrobial activity. Total lipid analysis of 2CCA-1 treated bacteria identified unique masses that were modeled to 2CCA-1 containing lysophosphatidic and phosphatidic acid in wild-type but not in *fakB3* mutant bacteria. This suggests that 2CCA-1 is metabolized as a fatty acid via FakB3 and utilized as a phospholipid building block, leading to accumulation of toxic phospholipid species. Analysis of FabT-mediated *fakB3* expression elucidates how the pneumococcus could ensure membrane homeostasis and concurrent economic use of host-derived fatty acids.

## Abstract (precis)

Fatty acid biosynthesis is an attractive antibiotic target, as it affects the supply of membrane phospholipid building blocks. In Streptococcus pneumoniae, it is not sufficient to target only the endogenous fatty acid synthesis machinery, as uptake of host fatty acids may bypass this inhibition.

## INTRODUCTION

Streptococcus pneumoniae (the pneumococcus) is a major contributor to morbidity and mortality globally, being the major cause of milder infections, such as otitis and sinusitis, and more severe respiratory tract infections, such as community-acquired pneumonia, sepsis, and meningitis. To cause infections, the bacteria first colonize the nasopharynx and from there spread to other organs. If pneumococcal colonization could selectively be targeted by narrow-spectrum antimicrobials, we could potentially reduce the pneumococcal disease burden in society, without harming the entire residing bacterial flora. Penicillin has been the first choice for treatment for decades, but resistance rates are increasing, threatening effective therapy (1). Therefore, new approaches are required, and novel bacterial targets need to be explored. Bacterial fatty acid metabolism has been identified as an attractive target for antimicrobial drug development due to its essentiality for cell membrane biosynthesis and its divergence from corresponding mammalian systems (2–5). The *de novo* fatty acid synthesis pathway (FASII) utilizes acetyl coenzyme A (acetyl-CoA) as a substrate to synthesize acyl chains on acyl-carrier proteins (ACP) with the combined action of enzymes encoded by the fatty acid synthesis operon (6, 7). Most Gram-positive bacteria utilize the PlsX/PlsY/PlsC pathway to catalyze the acylation of glycerol-3-phosphate and produce phosphatidic acid as a precursor for all phospholipid variants (8). Several inhibitors of enzymes in the FASII systems have been reported (2–5, 9). Such inhibitors might not be effective against pneumococci, as this species, like other bacteria belonging to the order *Lactobacillales*, can synthesize its membrane essentially entirely from extracellular, medium- or host-derived fatty acids and completely suppress their FASII system through transcriptional and biochemical inhibition (10–13).

Transcriptional repression of the *fab* operon, encoding enzymes of the FASII system, is mediated by the transcription factor FabT (14). FabT has increased affinity for DNA binding in the presence of a corepressor, such as the FASII operon-encoded acyl carrier protein 1 (ACP1; locus tag SPD_0381) that mediates feedback inhibition of the FASII system when acylated with long acyl chains (15). Also, recently the auxiliary acyl carrier protein 2 (ACP2; locus tag SPD_0044) was described to mediate FabT binding as a cofactor when acylated with extracellular fatty acids (16–18). Biochemical regulation of *de novo* fatty acid synthesis occurs at its first step through acetyl-CoA carboxylase (ACC) inhibition by an unknown ligand that is postulated to be either acyl-ACP or acyl-PO_4_ (11). In Gram-positive bacteria, such as pneumococci, extracellular fatty acids are activated by a fatty acid kinase (FakA) and by fatty acid binding proteins (FakB) to initiate phospholipid synthesis (16, 19, 20). Phosphorylated fatty acids (acyl-PO_4_) serve as substrates for PlsY for lysophosphatidic acid formation from glycerol-3-phosphate (G3P) or, after conversion by the acyl:PO_4_ transacylase PlsX to acyl-ACP, can be used as the substrate for PlsC to acylate G3P in position 2 for phosphatidic acid biosynthesis (21–23).

In body fluids, fatty acids are mainly bound to carrier proteins such as human serum albumin (24, 25), and its bovine orthologue is used in *in vitro* experiments to supplement bacterial growth with extracellular fatty acids (11, 26). However, free fatty acids can also exert antimicrobial activity, which is exploited by the innate immune system (27–29), interbacterial competition (30), and their application as antimicrobial agents (31–33). Antibacterial mechanisms of free fatty acids are species specific and diverse (31, 33), including membrane solubilization, alteration of membrane fluidity, and uncoupling of protein interactions, leading to impairment of energy production or nutrient transport (34), as well as membrane pore formation (26) or inhibition of *de novo* fatty acid synthesis enzymes (35).

In this study, we investigated the effects of the small-molecule compound 2CCA-1 and found that it induces a prominent autolytic and antimicrobial response in S. pneumoniae. Through analysis of resistant mutants, we identified 2CCA-1 as a fatty acid mimetic that is utilized by FakB3 to supply toxic building blocks for membrane phospholipid biosynthesis. The involvement of the transcriptional repressor of *de novo* fatty acid synthesis FabT in 2CCA-1 resistance revealed an additional role in modulation of *fakB3* expression as a response to extracellular fatty acid concentration. Our data suggest that 2CCA-1 may act as a narrow-spectrum antibacterial agent whose activity is dependent on a protein, found in S. pneumoniae and related species, required for extracellular polyunsaturated fatty acid metabolism (16).

## RESULTS

### Resistance to compound 2CCA-1 involves inactivation of either *fakB3* or *fabT*.

**FIG 1:**
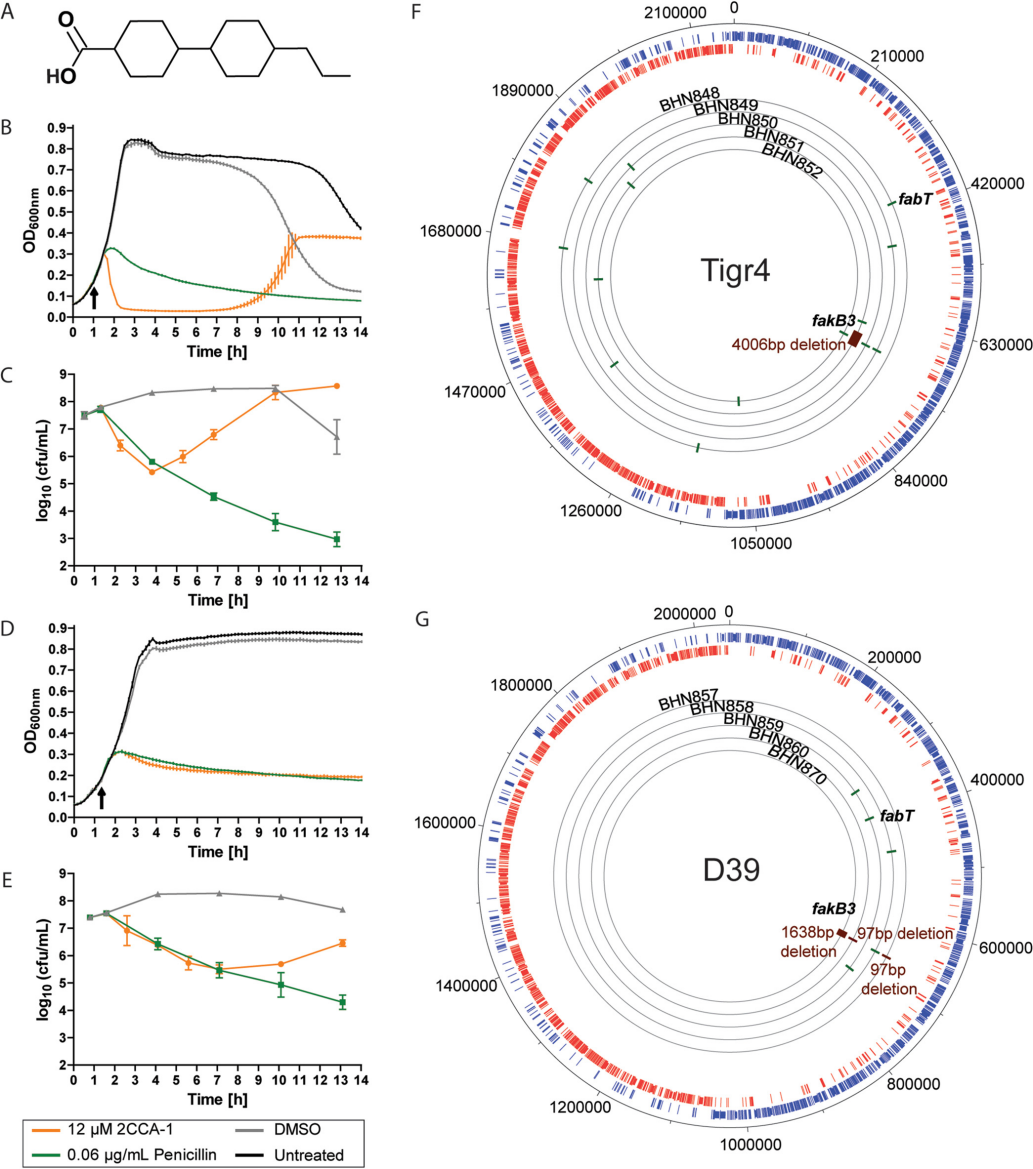
Characteristics of the alkylated dicyclohexyl carboxylic acid 2CCA-1. (A) Structure of 2CCA-1. (B and D) Optical density measurement and (C and E) viability determination characterizing the lysis curve of (B and C) S. pneumoniae D39 wild-type and (D and E) D39Δ*lytA* (grown in the presence of 110 mM choline chloride) treated with 2CCA-1 (12.5 μM) compared to penicillin (0.06 μg/ml) or DMSO (1% [vol/vol]) as the solvent control. Arrows indicate time of compound addition. Optical density measurements of a representative experiment are shown as averages ± SD from technical triplicates. Viability data are shown as averages ± SD from three biological replicates. (F) Overview of whole-genome sequenced 2CCA-1-resistant Tigr4 derivative strains (BHN848 to BHN852) displaying locations of single-nucleotide polymorphisms (green) and deletions (red) and (G) the respective D39 derivative 2CCA-1-resistant strains (BHN857 to BHN860 and BHN870). The observed genetic changes of the resistant strains highlighted are described in detail in Table 1.

We identified compound 2CCA-1 as a novel inducer of autolysin-mediated lysis in S. pneumoniae. The alkylated dicyclohexyl carboxylic acid 2CCA-1 comprises a carboxylic acid linked to a central dicyclohexyl scaffold, which in turn is connected to a short aliphatic chain (Fig. 1A). Pneumococcal cell wall hydrolases are essential for cell division and growth (36) but are also involved in various lysis phenomena of the pneumococcus, such as autolysis upon antibiotic-induced cell wall synthesis inhibition (37, 38). The initial pneumococcal response to early-logarithmic-phase treatment with 2CCA-1 resembled the response to penicillin, with induction of lysis about 1 h after challenge (Fig. 1B) and comparable death kinetics for the first 3 h (Fig. 1C). Furthermore, as for penicillin, cell wall hydrolases are involved in the lytic response of the pneumococcus upon 2CCA-1 treatment (Fig. 1D) and speed up the death process, even though bacterial death also occurs in the absence of cell wall hydrolase activity (Fig. 1E) (37–39).

Resistant mutants appeared following 2CCA-1 treatment, which restored logarithmic growth of the culture (Fig. 1B and C). Ten of the 2CCA-1-resistant mutants in the Tigr4 and D39 pneumococcal strain backgrounds were selected from four independent experimental pools and subjected to whole-genome sequencing; results are presented in Fig. 1F and G and complemented by a detailed description of the detected mutations in Table 1. Most of the resistant isolates (BHN849 to -852, BHN857 to -858, BHN860, and BHN870) were found to have mutations in the *fakB3* gene. These mutations included single nucleotide polymorphisms leading to stop codon mutations but also major deletions of 97 bp (BHN857 and BHN860), 1,638 bp (BHN870), and even 4,006 bp (BHN851) affecting the *fakB3* gene. This gene encodes a recently described fatty acid binding protein, FakB3, shown to be involved in the uptake of polyunsaturated fatty acids derived from the host environment for utilization in phospholipid biosynthesis in pneumococci (16). Two of the isolates, BHN857 and BHN860, had similar 97-bp deletions in *fakB3* and had no other mutation in comparison to the parental D39 strain. One Tigr4 mutant (BHN848) and one D39 mutant (BHN859) had an intact *fakB3* gene but instead contained mutations (a frameshift mutation and a nonsynonymous substitution) in the *fabT* gene, encoding the transcriptional repressor of the *fab* operon. Thus, these data imply that mutations inactivating either *fakB3* or *fabT* rescue pneumococci from the antibacterial effects of 2CCA-1.

**TABLE 1:** Overview of the genetic mutations found by whole-genome sequencing of mutants resistant to 2CCA-1^*a*^

Strain	Nucleotide mutation	Amino acid change	Locus tag	Protein function
BHN848	**A344-**	**Frameshift-stop codon**	**SP_0416**	**FabT, MarR family transcriptional regulator**
	A1601C	D534A	SP_1221	Type II restriction endonuclease
	G253-	Frameshift	SP_RS08765	Glycosyltransferase family 2 protein
	G992A	G331D	SP_1901	RNA methyltransferase

BHN849	C2030A	A677E	SP_0498	YSIRK-type signal peptide-containing protein
	**G808T**	E270*	**SP_0742**	**FakB3, DegV family protein**

BHN850	**T33G**	**C11W**	**SP_0742**	**FakB3, DegV family protein**
	T75C	N25N	SP_1493	MucBP domain-containing protein
	A1896491C			Noncoding region

BHN851	A1094T	D365A	SP_0686	Bacteriocin-associated integral membrane family protein
	**700052–704058; 4,006-bp deletion**		**SP_0737–SP_0742**
	G601T	A201S	SP_1706	Hypothetical protein
	G1821T	K607N	SP_1960	DNA-directed RNA polymerase subunit beta

BHN852	**A625T**	K209*	**SP_0742**	**FakB3, DegV family protein**
	G127A	E43K	SP_1136	DnaD domain protein

BHN857	**300–396; 97-bp deletion**		**SPD_0646**	**FakB3, DegV family protein**

BHN858	**T386A**	**V129E**	**SPD_0646**	**FakB3, DegV family protein**
	T222C	S74S	SPD_0453	Restriction endonuclease subunit S

BHN859	**A226G**	**K76E**	**SPD_0379**	**FabT, MarR family transcriptional regulator**
	C947T	A316V	SPD_0320	Glycosyltransferase family 1 protein
	C1169T	A390V	SPD_0709	DNA gyrase subunit B
BHN860	**300–396; 97-bp deletion**		**SPD_0646**	**FakB3, DegV family protein**
BHN870	**665175–666813; 1,638-bp deletion**	**Frameshift-Stop codon**	**SPD_0645-SPD_0646**	

a Boldface indicates genes or genetic regions affected by mutations in more than one resistant mutant.

Bioinformatic analysis of *fakB3* and *fabT* revealed that both genes are highly conserved and present in all the pneumococcal genomes sequenced so far. The nucleotide conservation of *fakB3* and *fabT* sequences among different serotypes and sequence types of pneumococci was investigated by using a BLAST search (40) against the pneumococcal PubMLST database (41) comprising 8,351 whole-genome sequences. The vast majority (99.6%) of the pneumococcal genomes possess a *fakB3* sequence that displays more than 98% similarity to the Tigr4 *fakB3* sequence. For *fabT*, 99.9% of the genomes possess a sequence that is more than 98% similar to the Tigr4 sequence. Further bioinformatic analyses revealed that FakB3 has homologues among the *Firmicutes* that are in the order *Lactobacillales* but is generally absent among species belonging to the order *Bacillales* (Fig. S1A), potentially explaining the low sensitivity to 2CCA-1 observed for Staphylococcus aureus (Fig. S1B).

10.1128/mBio.03027-20.4 FIG S1 FakB3 conservation in *Firmicutes* and S. aureus 2CCA-1 sensitivity. Download FIG S1, PDF file, 0.4 MB. Copyright © 2020 Reithuber et al. 2020 Reithuber et al. This content is distributed under the terms of the Creative Commons Attribution 4.0 International license.

**FIG 2:**
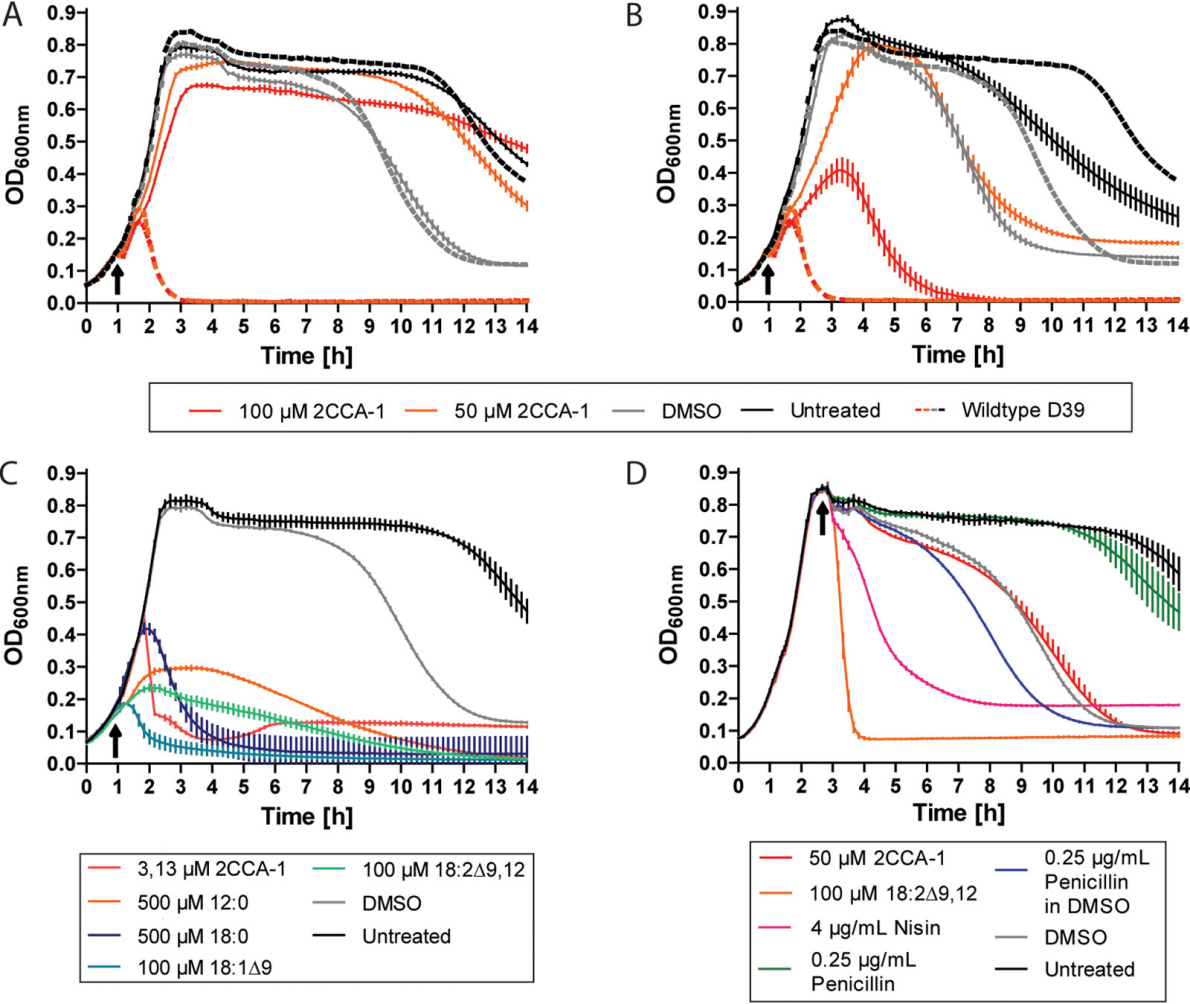
The fatty acid mimetic 2CCA-1 differs in the mode of action from antibacterial fatty acids. (A) D39Δ*fakB3* (BHN2024) and (B) D39Δ*fabT* (BHN2032) treated with 2CCA-1 (100 μM and 50 μM) and DMSO (1% [vol/vol]) as a compound solvent control in early logarithmic phase (arrows indicate time of treatment). Corresponding treatments of wild-type D39 are shown as averages for reference (dotted lines). (C) Comparison of the lysis inducing activity of free fatty acids of different saturation degree and acyl chain length with 2CCA-1 at their respective MlytC concentration on wild-type D39 (see also Fig. S6C to G) with lauric acid (12:0; 500 μM), stearic acid (18:0; 500 μM), oleic acid (18:1Δ9; 100 μM), linoleic acid (18:2Δ9,12; 100 μM), and 2CCA-1 (3 μM). (D) Treatment of wild-type D39 30 min after entering stationary phase with 2CCA-1 (50 μM; 16× MlytC), linoleic acid (18:2Δ9,12; 100 μM; 1× MlytC), nisin (4 μg/ml; 8× MIC), and penicillin dissolved in DMSO or water (0.25 μg/ml; 8× MIC).

Gene replacements of *fakB3* and *fabT* in the wild-type D39 background with antibiotic resistance genes (open reading frames [ORFs] or cassettes) confirmed that these genes are indeed required for the lytic response to 2CCA-1 treatment (Text S1; Tables S1 and S2; Fig. S2 to S4). D39Δ*fakB3* (BHN2024) did not respond with lysis upon even 100 μM 2CCA-1 treatment, and compared to the wild-type response, D39Δ*fabT* (BHN2032) showed reduced sensitivity to high concentrations of 2CCA-1 (Fig. 2A and B). Further validation was obtained by restoring the mutant alleles of *fakB3* and *fabT* in the spontaneous 2CCA-1-resistant isolates BHN857 and BHN859 with wild-type alleles, which reconstituted the wild-type sensitive phenotype to 2CCA-1 treatment (Fig. S5A to D). Two of the spontaneous resistant mutant strains (BHN851 and BHN870) contained large deletions that affected more genes than *fakB3*. Investigation of strains with constructed knockouts of these adjacent genes showed, however, that they did not contribute to the 2CCA-1-resistant phenotype (Fig. S5E to I). The preserved 2CCA-1 sensitivity of deletion mutants affecting the genes encoding the other two pneumococcal fatty acid binding proteins, *fakB1* (BHN2030) and *fakB2* (BHN2031), as well as their double deletion mutant (BHN1351), showed that FakB3 was the only pneumococcal fatty acid binding protein required for the lytic response to 2CCA-1 (Fig. S5J to L).

10.1128/mBio.03027-20.1 TEXT S1 Supplemental materials and methods. Download Text S1, PDF file, 0.2 MB. Copyright © 2020 Reithuber et al. 2020 Reithuber et al. This content is distributed under the terms of the Creative Commons Attribution 4.0 International license.

10.1128/mBio.03027-20.2 TABLE S1 Pneumococcal strains used in the study. Download Table S1, PDF file, 0.1 MB. Copyright © 2020 Reithuber et al. 2020 Reithuber et al. This content is distributed under the terms of the Creative Commons Attribution 4.0 International license.

10.1128/mBio.03027-20.3 TABLE S2 Primers used in the study. Download Table S2, PDF file, 0.2 MB. Copyright © 2020 Reithuber et al. 2020 Reithuber et al. This content is distributed under the terms of the Creative Commons Attribution 4.0 International license.

10.1128/mBio.03027-20.5 FIG S2 Sequential overlap PCR methodology for making transformation constructs. Download FIG S2, PDF file, 0.4 MB. Copyright © 2020 Reithuber et al. 2020 Reithuber et al. This content is distributed under the terms of the Creative Commons Attribution 4.0 International license.

10.1128/mBio.03027-20.6 FIG S3 Overview of the *fabT* operon in the wild-type and mutant strains produced in the study. Download FIG S3, PDF file, 0.5 MB. Copyright © 2020 Reithuber et al. 2020 Reithuber et al. This content is distributed under the terms of the Creative Commons Attribution 4.0 International license.

10.1128/mBio.03027-20.7 FIG S4 Overview of the *fakB3* locus and neighboring genes in the wild-type and mutant strains produced in the study. Download FIG S4, PDF file, 0.5 MB. Copyright © 2020 Reithuber et al. 2020 Reithuber et al. This content is distributed under the terms of the Creative Commons Attribution 4.0 International license.

10.1128/mBio.03027-20.8 FIG S5 2CCA-1 sensitivity of spontaneous and constructed pneumococcal mutants. Download FIG S5, PDF file, 0.8 MB. Copyright © 2020 Reithuber et al. 2020 Reithuber et al. This content is distributed under the terms of the Creative Commons Attribution 4.0 International license.

### 2CCA-1 possesses features of a fatty acid, indispensable for its antipneumococcal function.

The structure-activity relationship (SAR) of 2CCA-1 was explored by determining a minimal lytic concentration (MlytC) for related structural variants (Table 2). The MlytC was defined as the minimal concentration required to induce lysis during logarithmic growth of S. pneumoniae D39 grown in supplemented casitone and yeast extract (C + Y) medium. This measure facilitated a comparative analysis of the initial lysis-inducing capacity of structural variants which would have been obscured in a conventional MIC assay due to resistance development. The importance of carboxylic acid functionality for the activity of 2CCA-1 (MlytC of 3 μM) was highlighted by changing the carboxylic acid to a primary carboxamide, which resulted in analogue 2, with a MlytC of >100 μM (Fig. 1A and Table 2). Also, compounds 3 and 4, where the carboxylic acid had been changed to an acetate and a morpholine amide, respectively, showed a significant reduction of activity. These two analogues also had the first cyclohexyl ring changed to a phenyl ring, and these changes resulted in a clear decrease in activity. In addition, the presence of two connected cyclohexyl groups in position RII was found to be essential for activity, since compound 5, with only one cyclohexyl group in RII, was inactive. Activity of 2CCA-1 was dependent on the saturation status of the dicyclohexyl motif of RII, since one or two unsaturated phenyl groups rendered compounds 6 and 7 inactive. The only modifications that were tolerated were extensions of the alkyl chain in position RIII, from a 3-carbon chain (in 2CCA-1) to a 4- or 5-carbon chain (compounds 8 and 9), which provided MlytC of 6 and 3 μM, respectively (Table 2). Further extension of the alkyl substituent to 7 carbons, as in compound 10, caused a reduction in activity (MlytC between 25 μM and 50 μM).

**TABLE 2:**
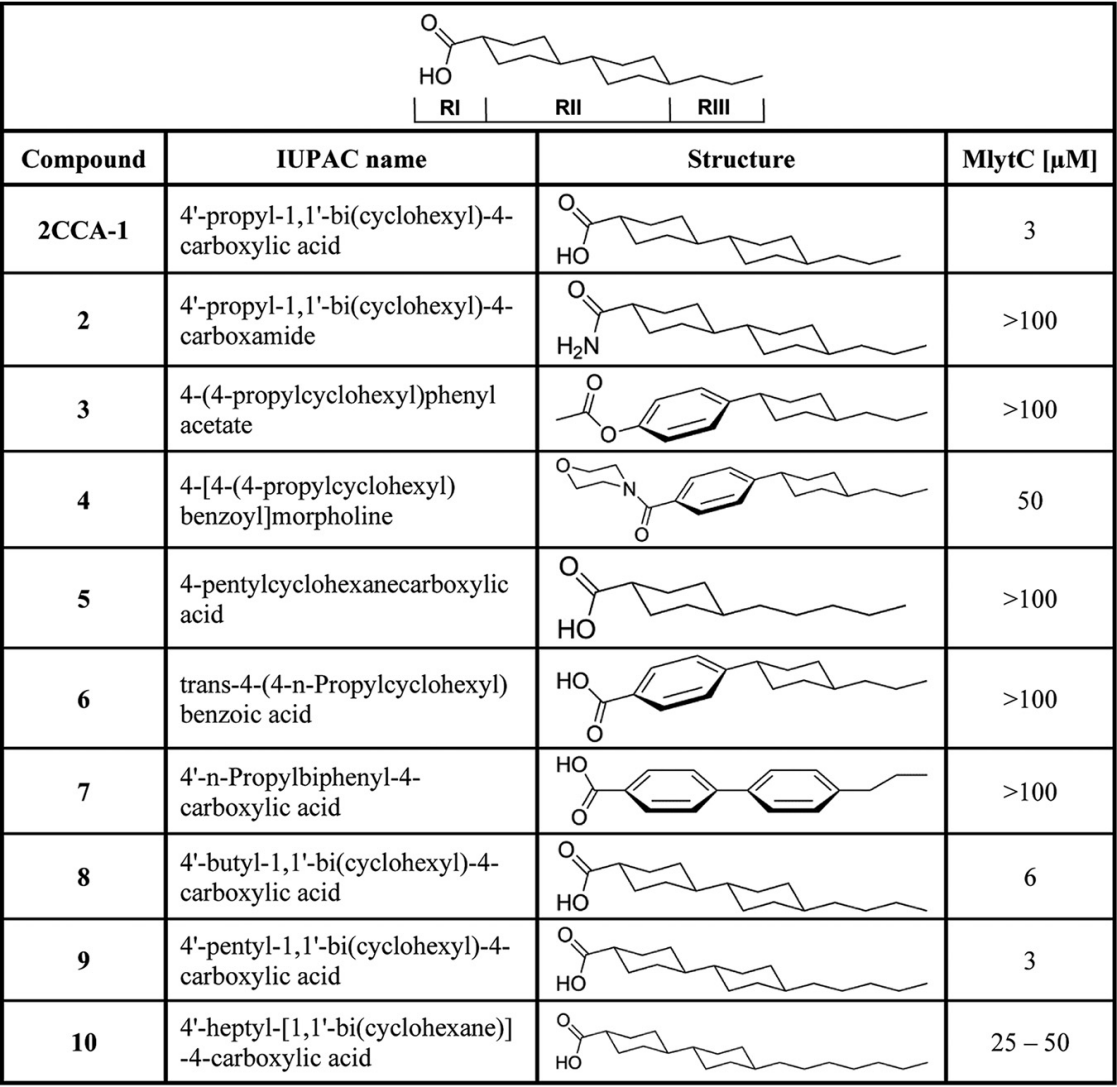
Structure-activity relationship (SAR) data

With this insight into SAR, we concluded that the structural integrity of 2CCA-1 was crucial for its antipneumococcal activity and only minor extensions of the alkyl chain in RIII were tolerated. With a carboxyl acid linked to a hydrophobic hydrocarbon scaffold, 2CCA-1 contains key features of a fatty acid. Indeed, examination of the stereo conformation of 2CCA-1 in comparison with lauric acid, a saturated 12-carbon fatty acid, revealed an almost identical structure when 2CCA-1 was tilted 90° around the axis of its hydrophobic core (Fig. S6A and B).

10.1128/mBio.03027-20.9 FIG S6 The fatty acid mimetic 2CCA-1. Download FIG S6, PDF file, 2.1 MB. Copyright © 2020 Reithuber et al. 2020 Reithuber et al. This content is distributed under the terms of the Creative Commons Attribution 4.0 International license.

### 2CCA-1 exerts its antibacterial effect after metabolization to form acyl chains of phospholipids.

The structural features of 2CCA-1 and the involvement of fatty acid metabolism proteins in resistance to 2CCA-1 suggested that the compound interacts with the bacteria as a fatty acid. Free fatty acids can exert antibacterial activity by solubilizing the bacterial membrane in a surfactant-like manner (26, 31, 33). However, in the case of 2CCA-1, several observations argued against such a direct mode of action. For one, 2CCA-1 activity was dependent on functional FakB3 or FabT, since in their absence, pneumococci were resistant to high concentrations of 2CCA-1 (Fig. 2A and B). Furthermore, the lysis-inducing capacity of 2CCA-1, judged by MlytC (Fig. 2C; Fig. S6C to G), was markedly more prominent than that of a panel of free fatty acids (2CCA-1, MlytC = 3 μM; lauric acid (12:0), MlytC = 500 μM; stearic acid (18:0), MlytC = 500 μM; oleic acid (18:1Δ9), MlytC = 100 μM; and linoleic acid (18:2Δ9,12), MlytC = 100 μM). In addition, 2CCA-1 did not induce lysis of cultures in stationary phase even at a concentration 16 times higher than the MlytC (Fig. 2D). This implies that 2CCA-1, like penicillin, needs actively growing cells for exerting its antibacterial activity.

Stationary-phase treatment with linoleic acid at its MlytC and with membrane-active antibiotics such as nisin (lipid II binding and pore forming) (42) and daptomycin (lipid II binding and membrane microdomain targeting) (43, 44) (Fig. S6H) induced a rapid lysis clearly distinguishable from the effect of the solvent dimethyl sulfoxide (DMSO) (Fig. 2D). Combined, these observations did not support a direct membrane perturbation activity of 2CCA-1 and could instead imply a requirement of further metabolic processing of 2CCA-1 to elicit its activity.

**FIG 3:**
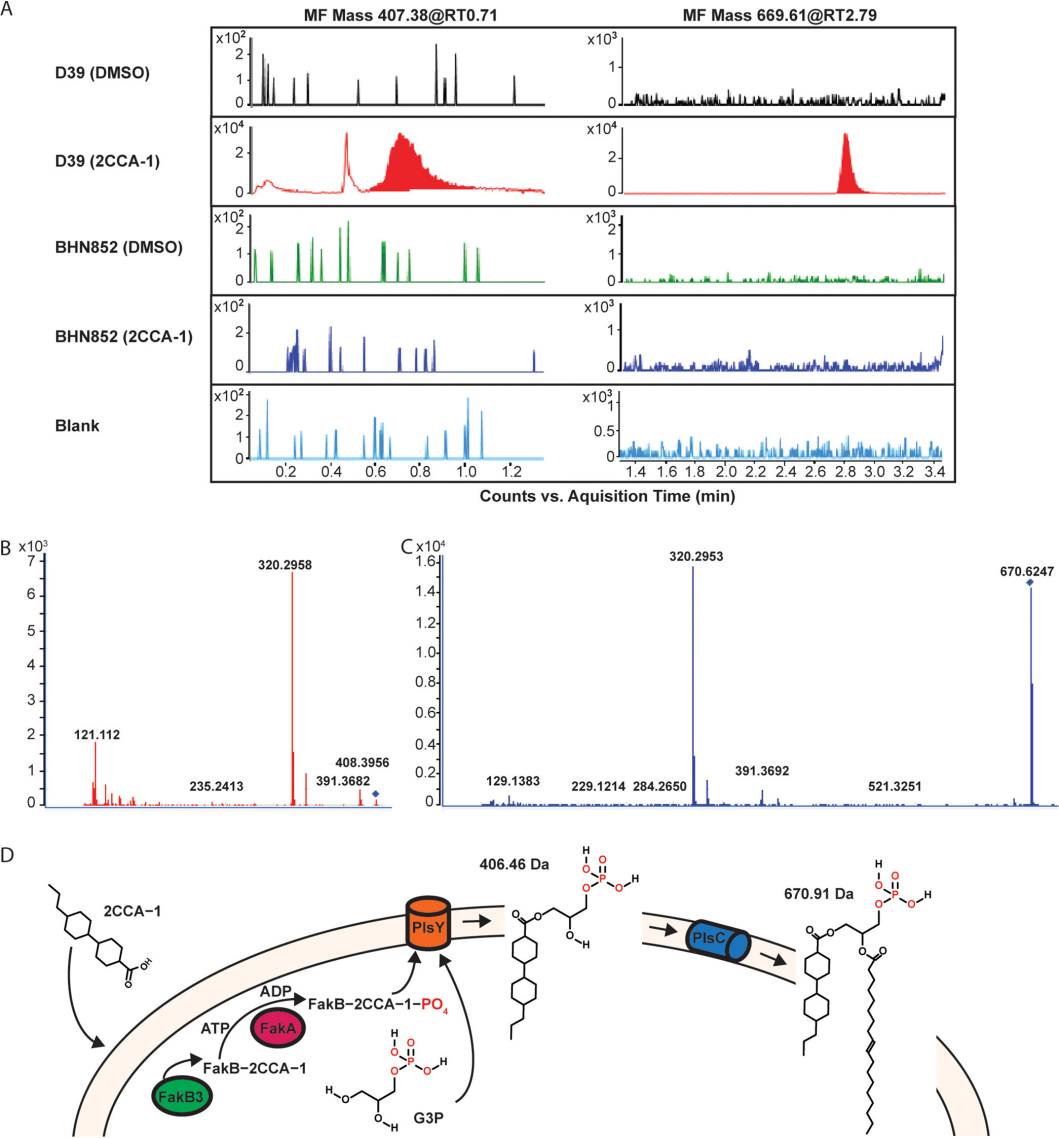
Lipidomic analysis of 2CCA-1 treated pneumococci. (A) Chromatograms showing molecular features (MF) 407.38 and 669.61 in wild-type Tigr4 in comparison with the *fakB3* mutant BHN852, when treated with 2CCA-1 (25 μM) or DMSO (1% [vol/vol]) as a solvent control. The prominent peaks of MF 407.38 at retention time (RT) 0.71 min and MF 669.61 at RT 2.79 min were found only in 2CCA-1-treated wild-type Tigr4 but were absent in the DMSO-treated Tigr4 sample as well as in the spontaneous resistant *fakB3* mutant BHN852. The color spikes in other samples are regarded as background noise (observe difference in scale [10^4^] in the 2CCA-1-treated wild-type Tigr4 sample). MS-MS spectra of (B) MF 407.38, RT 0.71 min, and (C) MF 669.61, RT 2.79 min, show similar fragments (320.29 and 391.36), indicating similar molecular structures. (D) Proposed model for 2CCA-1-mediated toxicity. 2CCA-1 associates with the pneumococcal plasma membrane, where it is taken up by FakB3 and gets phosphorylated by FakA, to become a substrate for PlsY. PlsY acylates 2CCA-1 onto glycerol-3 phosphate (G3P) to form lysophosphatidic acid (with a calculated mass of 406.46 Da). The subsequent addition of an 18 carbon unsaturated fatty acid by PlsC forms a 2CCA-1-containing phosphatidic acid (with a calculated mass of 670.91 Da).

As pneumococci use extracellular fatty acids solely as building blocks for membrane lipids and not for β-oxidation (8), we performed total lipid analysis (lipidomics) to see if we could identify a metabolic product of 2CCA-1. Indeed, two unique molecular features (MF) (mass [in Da] 669.61, retention time [RT, in minutes] 2.7; mass 407.38, RT 0.7) were identified in 2CCA-1-treated wild-type Tigr4 cells (Fig. 3A; Data Set S1). These two molecular features were absent in DMSO-treated wild-type Tigr4 cells and in the 2CCA-1-resistant strain BHN852 (97-bp deletion in *fakB3*) when treated with 2CCA-1 or DMSO. A very small unique peak was identified for MF 992.55 at RT 2.8 in the presented experiment, but only MF 407.38 (RT 0.7) and MF 669.61 (RT 2.7) were repeatedly identified in independent experiments. A LIPID MAPS search did not match these masses to any known lipid. The tandem mass spectrometry (MS-MS) spectra of MFs 669.61 and 407.38 both contained fragments of 320.29 Da and 391.36 Da, suggesting that they may share a similar structure and/or belong to the same pathway (Fig. 3B and C).

10.1128/mBio.03027-20.10 DATA SET S1 Lipidomics data set. Download Data Set S1, XLSX file, 0.1 MB. Copyright © 2020 Reithuber et al. 2020 Reithuber et al. This content is distributed under the terms of the Creative Commons Attribution 4.0 International license.

Modeling of 2CCA-1 as an acyl chain of lysophosphatidic acid and with phosphatidic acid in combination with an 18 carbon unsaturated fatty acid attached as a second acyl group, yielded theoretical fully protonated products of 406.46 Da and 670.91 Da, respectively (Fig. 3D), correlating with the unique masses obtained by lipid analysis of 2CCA-1-treated wild-type cells. Based on these findings, we propose a model where 2CCA-1 is taken up by FakB3 and is further metabolized as an extracellular fatty acid to form an acyl chain of phospholipids (Fig. 3D). The recorded dose-dependent changes in membrane fluidity upon 2CCA-1 treatment (Fig. S6I) could, in line with the proposed model and the observed autolysis induction, indicate a detrimental effect of the 2CCA-1-containing phospholipids on cell membrane-associated functions.

### The requirement of FabT for 2CCA-1 susceptibility suggests a regulatory interplay between FASII and FakB3-dependent extracellular fatty acid metabolism.

Pneumococcal resistance to 2CCA-1 in the absence of FakB3 or FabT suggests a functional link between these proteins. Indeed, a previous report analyzing genes regulated by FabT identified, besides 81 downregulated genes, four genes that were upregulated (14). Of these, *fakB3* (SP0742) was found to be 3.4-fold downregulated in the absence of FabT, suggesting a positive influence on expression by this transcription factor (14). However, FabT does not modulate *fakB3* expression through direct binding to the *fakB3* promoter (Fig. S6J to L).

**FIG 4:**
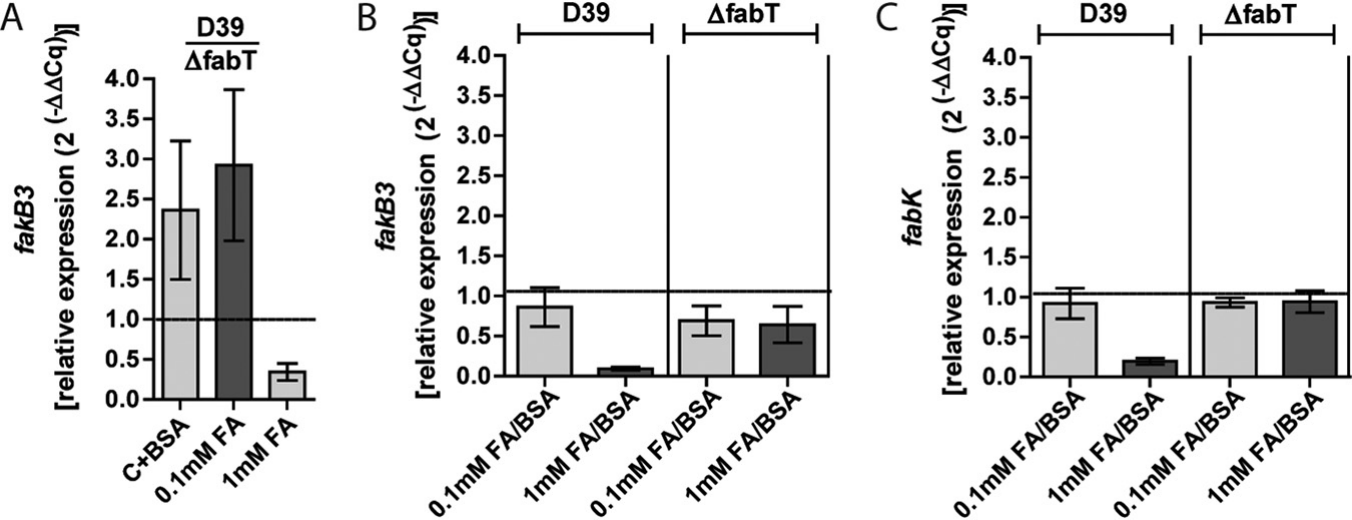
Role of FabT in 2CCA-1 resistance. (A) Relative *fakB3* expression in wild-type D39 compared to D39Δ*fabT* after growth in medium with fatty acids (0.1 mM or 1 mM) added to C medium with fatty acid-free BSA (10 mg/ml). (B) Relative *fakB3* expression in wild-type D39 and D39Δ*fabT* in medium with fatty acids (0.1 mM or 1 mM) added to C medium with BSA (10 mg/ml) compared to the expression in medium without added fatty acids. (C) Relative *fabK* expression in wild-type D39 and D39Δ*fabT* in medium with fatty acids (0.1 mM or 1 mM) added to C medium with BSA (10 mg/ml) compared to the expression in medium without added fatty acids. Relative gene expression (2^(−ΔΔ^*^Cq^*^)^) is shown as average ± SD of three biological replicates.

To further investigate the interaction of FabT and *fakB3*, we conducted transcription analyses under conditions of various extracellular fatty acid concentrations. Indeed, *fakB3* expression was found to be upregulated 2.36 ± 0.70-fold to 2.92 ± 0.77-fold (average ± standard deviation [SD]) in wild-type D39 (BHN853) in relation to D39Δ*fabT* (BHN2032) when grown in medium containing only BSA or a low concentration of 0.1 mM extracellular fatty acids, respectively (Fig. 4A). These data confirmed that the presence of FabT enhances transcription of *fakB3*. However, in the presence of higher concentrations of extracellular fatty acids (1 mM equimolar mixture of 18-carbon-chain fatty acids of different saturation degree), *fakB3* expression in wild-type D39 was 11.13 ± 1.62-fold (average ± SD) lower than in medium with fatty acid-free bovine serum albumin (BSA) and 9.27 ± 0.71-fold lower than in medium with low concentrations (0.1 mM) of fatty acids (Fig. 4B). This decrease in *fakB3* expression in medium containing 1 mM extracellular fatty acids was not observed in D39Δ*fabT* (BHN2032) (Fig. 4B), and under those conditions, *fakB3* was expressed at 3.07 ± 0.70-fold (average ± SD) lower levels in wild-type D39 than in D39Δ*fabT* (BHN2032) (Fig. 4A). This correlated with previous observations where a rigorous *fab* operon repression was found at high concentrations of extracellular fatty acids (10, 11). We confirmed that under the conditions investigated, *fabK* expression, as a readout for the *fab* operon regulated by FabT, was downregulated 5.25 ± 0.98-fold (average ± SD) for wild-type D39 grown in medium with 1 mM extracellular fatty acids compared to medium with BSA and 4.75 ± 0.76-fold compared to medium with 0.1 mM fatty acids. (Fig. 4C). The influence of FabT on *fabK* repression was also seen by the lack of differential expression in response to extracellular fatty acids in the Δ*fabT* strain (Fig. 4C). Indeed, *fabK* expression was 10.13 ± 0.41-fold (average ± SD) lower in wild-type D39 than in D39Δ*fabT* (BHN2032) in response to a high concentration (1 mM) of extracellular fatty acids. Thus, at high extracellular fatty acid concentrations, FabT not only acts as a transcriptional repressor of *fabK* but also exerts a repressive effect on *fakB3* (Fig. 4).

## DISCUSSION

In the present study, we describe a novel small antimicrobial compound, 2CCA-1, that potently induces autolysin-mediated pneumococcal lysis. Our data suggest a new antibacterial mechanism where 2CCA-1 functions as a fatty acid mimetic that does not primarily act as an anionic detergent on the pneumococcal membrane but becomes antibacterial after being metabolized and incorporated into phospholipids. Membrane fluidity is affected, likely by the cyclohexyl-containing structure, in combination with a relatively short alkyl chain of the resulting 2CCA-1-containing lipid. Alteration of membrane fluidity has been described to interfere with cell wall synthesis (44, 45), impairment of which is known to induce autolysis (37, 38), offering a plausible explanation for the induction of pneumococcal autolysis upon 2CCA-1 treatment, even though the compound does not directly target cell wall synthesis. Pneumococcal growth restriction was observed previously when cells were fed with extracellular saturated fatty acids (46). Accumulation of phospholipids with saturated acyl chains reduces membrane fluidity and eventually restricts growth (46). 2CCA-1 resembles in length the saturated middle chain lauric acid, which has long been known as the most potent antibacterial free fatty acid for Gram-positive bacteria (32). 2CCA-1 is, however, 160 times more potent than lauric acid in terms of lysis-inducing capacity (MlytC), suggesting that an accumulation of even minute amounts of 2CCA-1 in phospholipids can be detrimental for membrane homeostasis. However, with the data obtained so far, we cannot exclude the possibility that 2CCA-1 additionally inhibits enzymes of the FASII system, as described for linoleic acid (35).

**FIG 5:**
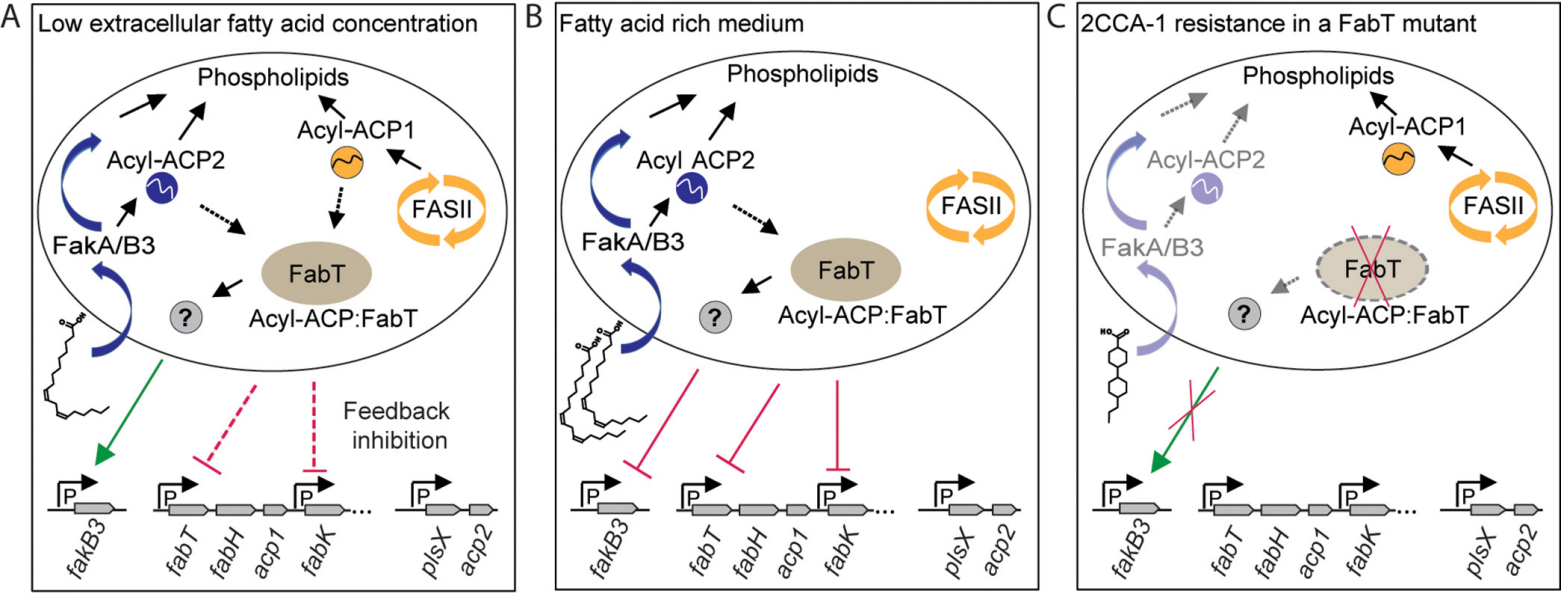
Proposed model for the regulatory interplay between the exogenous and endogenous fatty acid supply for phospholipid biosynthesis and its consequences for 2CCA-1 resistance. (A) Under conditions of low extracellular fatty acid availability, *fakB3* expression is enhanced by the presence of FabT. Since FabT does not bind to the *fakB3* promoter, the regulation is likely conferred indirectly by yet-unknown mediators. The main supply of phospholipid building blocks comes from the FASII system, which is subjected to feedback inhibition by FabT with long-chain acyl ACP1 as the corepressor (15). (B) Upon availability of abundant extracellular fatty acids, FabT:acyl-ACP2 mediates the repression of the *fab* operon (16–18). FabT also influences the repression of *fakB3*, resulting in decreased incorporation of polyunsaturated fatty acids (16) into the membrane, which could ensure its homeostasis. How *fakB3* repression is influenced by FabT remains to be identified. (C) Mutants with deletions of *fakB3* are resistant to 2CCA-1. A deleterious mutation in *fabT* abolishes the enhanced transcription of *fakB3* in medium with a low extracellular fatty acid concentration. Thus, in a *fabT* mutant, 2CCA-1 incorporation by *fakB3* could be greatly reduced, so that the mutant resists higher 2CCA-1 concentrations than a wild-type pneumococcal strain.

Examination of mutants resistant to 2CCA-1 revealed that they had inactivating mutations in two genes involved in fatty acid metabolism; these genes encode FakB3, a polyunsaturated fatty acid binding protein (16), and the transcription factor FabT. Investigation of these resistant mutants uncovered a functional link between FakB3 and FabT and revealed another layer of regulation of fatty acid supply for phospholipid synthesis, as proposed in Fig. 5. Under conditions of low fatty acid availability (Fig. 5A), *fakB3* expression was higher in the presence than in the absence of FabT. Upon availability of abundant extracellular fatty acids, however, *fakB3* expression was lower in the presence than in the absence of FabT (Fig. 5B). Indeed, repression of *fakB3* expression upon long-chain polyunsaturated arachidonic acid stress has also been observed previously (47). In the wild-type strain, *fakB3* expression was in general lower in medium with high concentrations than low concentrations of extracellular fatty acids (Fig. 4). Availability of FakB3 at low extracellular fatty acid concentrations could ensure the economic use of all accessible extracellular building blocks for cell membrane synthesis to disburden the energy-intensive endogenous fatty acid synthesis (12), which operates concurrently and is subjected to feedback inhibition only by long-chain acyl-ACP1 (14, 15). The decrease of *fakB3* expression and the resulting decrease of polyunsaturated fatty acid incorporation in the membrane could be a way to tightly control their content in the membrane for maintenance of growth-permissive membrane fluidity. S. pneumoniae synthesizes saturated and monounsaturated, but not polyunsaturated, fatty acids through the FASII system (11, 46–48). In the presence of extracellular fatty acids, FASII is metabolically and transcriptionally downregulated, and pneumococci can build up their membrane almost entirely from extracellularly supplied oleic acid (11). Equipped with three FakB homologues with affinity for all degrees of fatty acid saturation, the pneumococcus can scavenge the whole spectrum of human serum fatty acids (16). This, however, also requires an economic but tight control (12) of polyunsaturated fatty acid incorporation, influenced by FabT in response to explicit extracellular fatty acid concentration. Control of polyunsaturated fatty acid concentration could additionally be administered by sensors of membrane homeostasis, similarly to the regulation of fatty acid biosynthesis by the response regulator YycF of the pneumococcal two-component system YycFG (49, 50), or the global regulator CcpA in Streptococcus mutans (51).

In conclusion, we find that 2CCA-1 functions as a fatty acid mimetic and is the first reported agent that targets the extracellular fatty acid supply of the lipid biosynthesis in S. pneumoniae. Our data suggest that 2CCA-1, by interacting with the polyunsaturated fatty acid binding protein FakB3, becomes incorporated as novel phospholipid species in the bacterial membrane with antibacterial consequences. S. aureus, a representative of the order *Bacillales* lacking FakB3, displayed low 2CCA-1 sensitivity, indicating that the diversity in the host fatty acid metabolism among species of the phylum *Firmicutes* potentially provides targets for narrow-spectrum antibiotics. Furthermore, FabT, the repressor of the *fab* operon, was found to influence the expression level of *fakB3* in an intricate manner in response to extracellular fatty acid conditions. 2CCA-1 may serve as a valuable tool for investigating various aspects of pneumococcal lipid metabolism and membrane homeostasis, but it could also potentially form the basis for development of narrow-spectrum antimicrobials, a possibility which needs to be explored in future studies.

## MATERIALS AND METHODS

### Bacterial growth conditions.

S. pneumoniae strains (Table S1) were grown on blood agar plates over night at 37°C and 5% CO_2_. The standard medium for suspension cultures was C+Y medium (Text S1) supplemented with horse serum (1% [vol/vol]; Håtunalab) and glucose bouillon (9% [vol/vol], 25 g/liter nutrient broth no. 2 [Oxoid], 10 g/liter glucose). To assess the contribution of the pneumococcal cell wall-degrading enzymes to pneumococcal lysis, the bacteria were grown in medium containing a competitive concentration of choline chloride (110 mM) (Sigma). For establishing growth conditions without extracellular fatty acids, the yeast extract was omitted from the C+Y medium, yielding C medium. For experiments with defined fatty acid compositions, the C medium was supplemented with fatty acid-free BSA (10 mg/ml; Sigma) and with the appropriate fatty acids (lauric, stearic, oleic, and linoleic acids, purchased from Sigma) to a final solvent concentration of 1% (vol/vol) DMSO in the growth medium.

### Chemicals and antibiotics.

The following chemical compounds used in this study were purchased from ChemBridge: 2CCA-1 [4′-propyl-1,1′-bi(cyclohexyl)-4-carboxylic acid (C_16_ H_28_ O_2_); ChemBridge no. 5306127], compound 2 [4′-propyl-1,1′-bi(cyclohexyl)-4-carboxamide (C_16_H_29_NO); ChemBridge no. 5301965], compound 3 [4-(4-propylcyclohexyl)phenyl acetate (C_17_ H_24_ O_2_); ChemBridge no. 5536706], compound 4 {4-[4-(4-propylcyclohexyl) benzoyl]morpholine (C_20_ H_29_ NO_2_); ChemBridge no. 5562176], compound 5 [4-pentylcyclohexanecarboxylic acid (C_12_H_22_O_2_); ChemBridge no. 5303499], compound 8 [4′-butyl-1,1′-bi(cyclohexyl)-4-carboxylic acid (C_17_H_30_O_2_); ChemBridge no. 5304107], and compound 9 [4′-pentyl-1,1′-bi(cyclohexyl)-4-carboxylic acid (C_18_ H_32_ O_2_); ChemBridge no. 5309126]. Compound 10 {4′-heptyl-[1,1′-bi(cyclohexane)]-4-carboxylic} acid (C_20_H_36_O_2_); MolPort-002-798-661} was purchased from MolPort. Compound 6 [*trans*-4-(4-*n*-propylcyclohexyl) benzoic acid (C_16_H_22_O_2_)); H57552 and compound 7 (4′-*n*-propylbiphenyl-4-carboxylic acid [C_16_H_16_O_2_]; L06110) were purchased from Alfa Aesar. 2CCA-1 (Vitas-M no. STK058310) was also purchased from Vitas-M Laboratory.

2CCA-1 and its structural analogues, vancomycin hydrochloride (Sigma), and daptomycin cyclic lipopeptide antibiotic (Sigma) were dissolved in anhydrous DMSO (Invitrogen), nisin from Lactococcus lactis (2.5%, balance sodium chloride [Sigma]) was dissolved in acetic acid (0.05%), and penicillin G sodium salt (Fluka) was dissolved in water or DMSO as indicated.

### Assay for assessing the lysis inducing activity.

For investigating the lysis-inducing capacity of compounds, precultures of S. pneumoniae strains in supplemented C+Y medium were inoculated from overnight blood agar plates or frozen starter cultures. At mid-log phase, these precultures were diluted into fresh medium to an optical density at 600 nm (OD_600_) of ∼0.05 (∼2 × 10^7^ CFU/ml) and distributed into wells (400 μl per well) of a Honeycomb plate (Oy Growth Curves AB Ltd., Helsinki, Finland). Growth kinetics were monitored using a Bioscreen C plate reader (Oy Growth Curves AB Ltd.). Cultures were challenged in early log phase (OD_600_ ≈ 0.15; ∼3.3 × 10^7^ CFU/ml) with 2CCA-1, antibiotics, fatty acids, or other chemical compounds by adding 4 μl from a 100× stock solution, followed by mixing of the well contents. Thus, for compounds dissolved in DMSO, the final solvent concentration was 1% (vol/vol), which was also added as control treatment. For viability determination, samples were taken at timed intervals and the number of CFU was determined in appropriate dilutions after overnight incubation on blood plates. Viability after compound exposure was determined in triplicate biological experiments that were run in at least technical duplicates.

For determination of the minimal lytic concentration (MlytC) of 2CCA-1, SAR analogues, and free fatty acids, the above-described procedure was performed using the S. pneumoniae D39 strain grown in supplemented C+Y medium. When required, the spectrophotometric contribution of the chemicals or fatty acids was subtracted from the measured OD values, in addition to those of the uninoculated growth medium. The MlytC was defined as the lowest concentration that induced lysis in S. pneumoniae D39 before reaching an OD_600_ of 0.5 (∼1.24 × 10^8^ CFU/ml) under the described conditions. The investigations were carried out in technical triplicates and at least two biological replicates.

### Isolation of spontaneous resistant mutants and whole-genome sequencing.

Spontaneous 2CCA-1-resistant strains were isolated when 2CCA-1 (25 μM) was added to cultures of S. pneumoniae D39 or Tigr4 in their logarithmic growth phase as for the determination of lysis-inducing activity described above. Mutant strains BHN848 to BHN850 as well as BHN857 to BHN859 were isolated on blood agar plates from samples plated after 2 h compound exposure, and BHN851, BHN852, BHN860, and BHN870 were isolated from samples plated after overnight compound exposure (Table S1). Genomic DNA was isolated using Genomic-tip 100/G columns (Qiagen) following the manufacturer’s instructions with the following exceptions: strains were isolated directly from a blood agar plate and were incubated for 30 min at 37°C in buffer B1 with LytA (50 μg/ml), recombinantly produced as previously described (37), followed by a continued 20-min incubation at 37°C with proteinase K at the recommended concentration. Whole-genome sequencing of these spontaneous resistant mutants along with the parental wild-type strains was performed using TruSeq DNA library preps and Illumina Miseq V3, 2× 300 bp with a minimum of 100× coverage per base. The reads were quality checked using FastQC (52) and trimmed using Trimmomatic (53). All the reads were aligned to the respective reference genomes from NCBI (Tigr4, NC_003028.3; D39, NC_008533.2) using bwa (54). The variants were identified using SAMtools, GATK tools, and BEDTools (55–57) and visualized with DNAplotter (58).

### Construction of pneumococcal mutant strains.

Pneumococcal mutant strains were generated in which the open reading frames (ORFs) of the target genes were deleted and replaced with either antibiotic resistance ORFs or cassettes with promoter, ORF, and terminator sequences, as indicated in Table S1. A detailed description of how transformation constructions were generated is included in Text S1 as well as in Table S2 and Fig. S2 to S4.

### Stationary-phase lysis.

For the examination of lysis inducing activity of substances in stationary phase, suspension cultures of S. pneumoniae D39 were prepared as for the characterization of the lysis-inducing activity in logarithmic phase, but treatments were administered followed by mixing of the well contents after the bacteria had stayed in stationary phase for 30 min. Treatments consisted of 2CCA-1 (50 μM), linoleic acid (100 μM), nisin (4 μg/ml), and penicillin (0.25 μg/ml) dissolved in DMSO as well as in water, as well as daptomycin (8 μg/ml). For examining the effect of daptomycin, Ca^2+^ (50 μg/ml) from CaCl_2_ (Merck) was used to supplement the growth media. Treatments were carried out in triplicate and repeated at least twice. For OD values of 2CCA-1 treated bacteria, the spectrophotometric contribution of the chemical was subtracted.

### Lipid extraction, liquid chromatography, and mass spectrometry.

Bacteria (BHN842 and BHN852) were cultured as for experiments characterizing the lysis-inducing activity and treated with 2CCA-1 (25 μM) and DMSO (1% [vol/vol]) as a solvent control. When the cultures decelerated their logarithmic growth and started to lyse, a sample (400 μl) was immediately placed on ice to decelerate autolysis and centrifuged at ≥6,200 × *g* for 2 min at 4°C. The pellet was washed twice, once each with 1 ml and 500 μl NaCl (150 mM). The pellet was stored at −80°C until analysis. Total lipids from frozen pneumococcal cells were extracted using a chloroform-methanol (2:1 [vol/vol]) mixture and analyzed by ultra-high-performance liquid chromatography coupled with tandem mass spectrometry, as described in detail in Text S1. The strategy for data analysis was built on the hypothesis that wild-type Tigr4 metabolizes 2CCA-1 and produces hypothetical lipid compounds. Thus, we compared wild-type Tigr4 cells to BHN852 cells treated with 2CCA-1 or with 1% DMSO. Initially, metabolites resulting from untargeted lipidomics were graded using the following criteria: (i) a metabolite should be absent from a blank sample or be at least five times higher under an experimental condition than in blanks; (ii) the relative standard deviation (RSD%) of the area under the curve within an experimental condition (between 6 repeats) should not be more than 100%; (iii) the average area under the curve of the peak between at least 2 experimental conditions should differ at least two times; (iv) metabolites that met the first three criteria were analyzed using principal-component analysis (PCA), orthogonal partial least-square discriminant analysis (OPLS-DA), and variable influence on projection (VIP) by SIMCA (Sartorius), and metabolites important for separation of samples from different experimental conditions were selected at this stage (VIP score ≥ 1); (v) finally, visual inspection of peaks/chromatograms of selected compounds was done in Profinder (Agilent). From 3,636 molecular features (MFs) measured by untargeted lipidomics, we selected 14 that met the criteria of the hypothesis and the strategy. After the experiments were repeated 3 times, only one candidate MF still met the criteria, and one more MF was found by analogy and mere visual inspection. In all validation experiments, only MFs that were completely absent from all blank samples and present in all replicas of a certain experimental condition were included in the analysis.

### Determination of gene expression.

Single colonies of the investigated mutants and their isogenic wild-type strain (D39 [BHN853], D39Δ*fakB3*::*ermB* [BHN2024], and D39Δ*fabT*::*ermB* [BHN2032]) were propagated on blood plates and subsequently in liquid cultures of C medium with fatty acid-free BSA (10 mg/ml) and a 0.1 mM or 1 mM fatty acid mixture containing equimolar proportions of oleic, stearic, and linoleic acid. When the precultures reached mid-log phase, they were diluted in fresh medium to an OD_600_ of ∼0.05 and harvested when they reached an OD_600_ of ∼0.15 by immediate transfer to ice. Following centrifugation at ≥2,700 × *g* for 15 min. the pellets were taken up in 1 ml RNAPro solution from the FAST RNA Pro Blue kit (MP Biomedicals), and RNA was isolated according to the manufacturer’s instructions.

DNase treatment for 10 μg or 20 μg nucleic acid was carried out in 100-μl or 200-μl reactions, respectively, with Turbo DNase (0.06 U/μl; Invitrogen), Turbo DNase buffer (1×; Invitrogen), and 0.2 U/μl SUPERaseIn RNase inhibitor (Thermo Fisher Scientific). The reaction mixture was incubated for 45 min at 37°C. Subsequently, an equal volume of phenol-chloroform–isoamyl alcohol (25:24:1; Sigma) was added and mixed by inversion in Phase Lock Gel Heavy tubes (5 Prime). Aqueous phase separation was performed by centrifugation at ≥17.000 × *g* for 15 min at 16°C. The aqueous phases were transferred to new tubes, and RNA was precipitated with an equal volume of isopropanol (Sigma) for 30 min at room temperature, whereupon RNA was collected by centrifugation at ≥17.000 × *g* for 30 min. After the RNA was washed with 70% ethanol, the pellet was air-dried briefly, and RNA was suspended in diethyl pyrocarbonate (DEPC)-treated water. RNA was quantified spectrophotometrically (Nanodrop, Invitrogen). DNA contamination assessment was performed by PCR, and RNA integrity was verified by 1.5% (TAE) agarose gel electrophoresis. cDNA was produced with a high-capacity cDNA reverse transcription kit (Applied Biosystems) according to the manufacturer’s protocol without RNase inhibitor from 400 ng of DNase-treated RNA per sample.

Quantitative PCR was performed with iTaq universal SYBR green supermix (Bio-Rad) in a reaction volume of 20 μl with 375 nM forward and reverse primer (Table S2) and 1 μl of the cDNA reaction mixture. Thermocycling was performed in a Bio-Rad CFX96 instrument with conditions recommended by the manufacturer. Primers were validated on a standard curve using D39 genomic DNA as a template over a 5-log concentration range for amplification with comparable efficiency (59) and repeatability in three independent experiments. The PCR efficiency (average ± standard deviation) for primer pairs targeting different genes was as follows: for *fakB3*, 83.27 ± 2.41; for *fabK*, 84.88 ± 1.06; and for *gyrA*, 83.37 ± 1.44. A nontemplate control (NTC) and a no-reverse-transcriptase (NRT) control were included, which both resulted in quantification cycle (*C_q_*) values with a difference of at least 11 relative to the *C_q_* values of the investigated samples (excluding *fakB3* expression in D39Δ*fakB3*). *C_q_* values were retrieved from the instrument, and Δ*C_q_* values were calculated using the gyrase housekeeping gene (SPD_1077). Relative gene expression (2^(−ΔΔ^*^Cq^*^)^) (60), comparing expression in different strains or media, is reported as averages and standard deviations from three biological experiments analyzed in technical triplicates.
