# Supplementary material for: The Bactericidal Fatty Acid Mimetic 2CCA-1 Selectively Targets Pneumococcal Extracellular Polyunsaturated Fatty Acid Metabolism
Source: mBio. 2020 Dec 15;11(6):e03027-20. doi: 10.1128/mBio.03027-20 (PMC7773995; doi:10.1128/mBio.03027-20)
Supplement: FIG S1 [file mBio.03027-20-sf001.pdf]

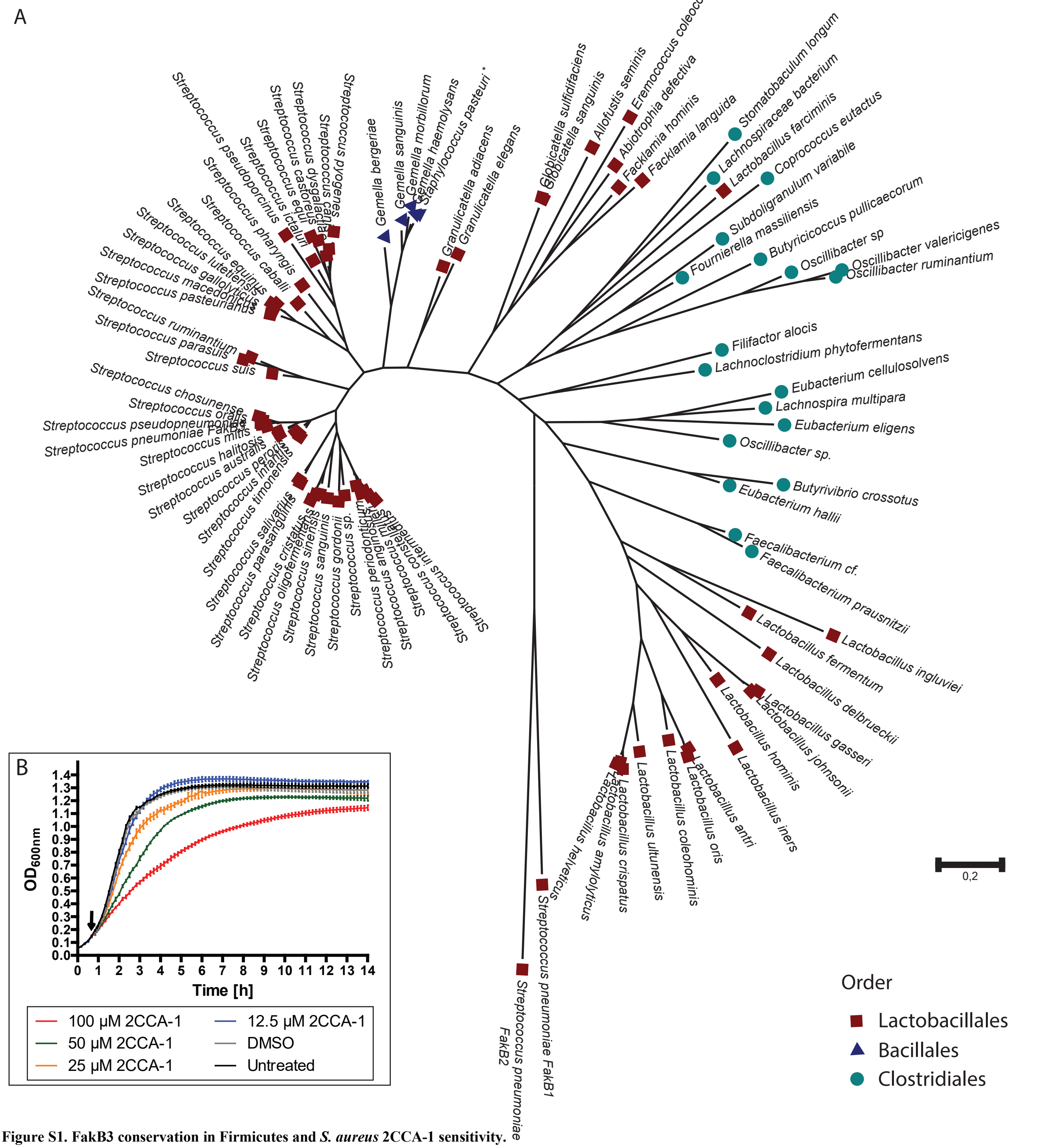

Figure S1. FakB3 conservation in Firmicutes and *S. aureus* 2CCA-1 sensitivity.
(A) Phylogenetic analysis of FakB3 homologues across Firmicutes. The evolutionary history was inferred by using the Maximum Likelihood method based on the JTT matrix-based model (Ref 1). The tree with the highest log likelihood (-26962,99) is shown. Initial tree(s) for the heuristic search were obtained automatically by applying Neighbor-Join and BioNJ algorithms to a matrix of pairwise distances estimated using a JTT model, and then selecting the topology with superior log likelihood value. The tree is drawn to scale, with branch lengths measured in the number of substitutions per site. The analysis involved 90 amino acid sequences. There was a total of 359 positions in the final dataset. Evolutionary analyses were conducted in MEGA7 (Ref 2). * For *Staphylococcus pasteuri,* only one strain in this species got the hit, where the other strains across this species lacks FakB3, indicating the strain might be incorrectly annotated. Extensive horizontal gene transfer between *Gemella* and *Streptococcus* genus has been described (Ref 3), potentially explaining *fakB*3 presence in the only genus of the Bacillales order. **(B)** Sensitivity of *S. aureus* to 2CCA-1 in supplemented BHI medium. Experimental conditions are described in the **Supplemental Materials and Methods**. Arrow indicate timepoint of treatment. Avg +/- SD of triplicate treatment in one representative biological experiment are shown.

Ref 1. Jones D.T., Taylor W.R., and Thornton J.M. (1992). The rapid generation of mutation data matrices from protein sequences. Computer Applications in the Biosciences 8: 275-282.
Ref 2. Kumar S., Stecher G., and Tamura K. (2016). MEGA7: Molecular Evolutionary Genetics Analysis version 7.0 for bigger datasets. Molecular Biology and Evolution 33:1870-1874.
Ref 3. García López E and Martín-Galiano AJ (2020) The Versatility of Opportunistic Infections Caused by Gemella Isolates Is Supported by the Carriage of Virulence Factors From Multiple Origins. Front. Microbiol. 11:524. doi: 10.3389/fmicb.2020.00524
