# Supplementary material for: The Bactericidal Fatty Acid Mimetic 2CCA-1 Selectively Targets Pneumococcal Extracellular Polyunsaturated Fatty Acid Metabolism
Source: mBio. 2020 Dec 15;11(6):e03027-20. doi: 10.1128/mBio.03027-20 (PMC7773995; doi:10.1128/mBio.03027-20)
Supplement: TEXT S1 [file mBio.03027-20-s0001.pdf]

Supplementary Materials and Methods

Composition of the C+Y medium:
Casamino acids (5 g/L), yeast extract (1.1 g/L), anhydrous sodium acetate (1.2 g/L), L-tryptophan (5 mg/L), L-cysteine (50 mg/L), L-glutamine (25 mg/L), sodium pyruvate (250 mg/L), sodium hydroxide (3 mM), glucose (455 mg/L), sucrose (56.9 mg/L), magnesium chloride (113.8 mg/L), calcium chloride dehydrate (0.894 mg/L), adenosine (4.55 g/L), uridine (4.55 g/L), asparagine (0.5 mg/L), choline chloride (50 µg/L), niacin (6 µg/L), pyridoxine hydrochloride (7 µg/L), D-pantothenic acid hemicalcium salt (24 µg/L), thiamine hydrochloride (6.4 µg/L), riboflavin (280 µg/L), biotin (0.6 µg/L), iron(II) sulfate heptahydrate (1.25 µg/L), copper(II) sulfate pentahydrate (1.25 µg/L), zinc chloride (0.6 µg/L), manganese(II)chloride tetrahydrate (0.55 µg/L), hydrochloric acid (3.7 ppm), potassium dihydrogen phosphate (281.3 mg/L), dipotassium hydrogenphosphate (6.169 g/L).

Construction of antibiotic resistance cassettes
Antibiotic resistance cassettes were constructed in which antibiotic resistance Orfs providing resistance to kanamycin and spectinomycin were PCR amplified and used to replace the *ermB* Orf in the previously described pSP72/Δ*lytA-erm* vector (1). In this vector the erm cassette consists of an *ermB* Orf flanked by a promoter and a terminator derived from vector pVA838 (2). The pSP72/Δ*lytA-erm* vector was PCR amplified with primers that included the promoter and terminator but omitted the *ermB* ORF. The *kanR* Orf and *spcR* Orf (encoding kanamycin and spectinomycin resistance proteins) were amplified with primers that contained overhang sequences complementary to the 5' and 3' ends of the amplified vector. A ligase-independent *in vivo* recombination procedure was used to join the fragments (1, 3, 4). The amplified vector was mixed with the amplified antibiotic resistance Orfs in separate tubes, treated with DpnI (New England Biolabs) for 3 h at 37 °C to degrade the methylated template DNA vector and 1 µL of the mixture was used to transform XL-gold competent cells (Agilent) (30 µL), where the vector and insert combine by homologous recombination. The vectors were isolated from clones after selection on kanamycin or spectinomycin-containing nutrient agar plates. The sequence of the resulting plasmids designated pSP72/Δ*lytA-kanR*-Cass and pSP72/Δ*lytA-spcR*-Cass were confirmed by Sanger sequencing. These vectors were used as templates to amplify the *kanR* and *spcR* cassettes, respectively. Thus, having the same promoter and terminator the *ermB*, *kanR* and *spcR* cassettes could be amplified using the same AbCass-Fr and AbCass-Re primer pair from the different vector templates.

Cloning of *fakB3* promoter into pPP2 vector and *fabT* into pET21d vector
The same *in vivo* recombination cloning method as mentioned in previous section was used to clone the promoter region of *fakB3* into the "Integrative promoter probe vector" pPP2 (GenBank Accession number EF061139.1) and to clone *fabT* into the pET21d vector (Merck) for protein expression. The pPP2 vector was PCR amplified as well as the 124 bp intergenic region upstream of *fakB3* start codon with primers given in **Table S1** and using genomic DNA isolated from *Streptococcus pneumoniae* strain D39 as a template. The primers used for the latter amplicon had overhang sequences complementary to the 3' and 5'-ends of the pPP2 amplicon. Likewise, the pET21d vector and the *fabT* Orf was amplified with specific primers (**Table S1**). The reverse primer of the *fabT* Orf (fabT-Re-6His-OH) and the forward primer of the pET21d vector (pET21-Fr-6His-OH) contained a non-annealing overhang sequence that included six histidine codons plus a TAA stop codon that were complimentary to each other and made up the interface for the in vivo recombination cloning procedure. Following PCR amplification, the vectors and the inserts were mixed at equimolar concentration. Following DpnI treatment to degrade the methylated template vector, the mixture was used to transform competent XL-gold as described above. The sequence of the isolated vectors, pPP2-PfakB3 and pET21d-fabT-6His, were confirmed by Sanger sequencing.

Construction of pneumococcal mutant strains

Transformation constructs were made by sequential extension overlap PCR according to the procedure described in **Fig. S2**. In a first step, using genomic DNA as a template (Genomic-tip 100/G kit (Qiagen)) (**Fig. S2A**) the regions immediately up and down-stream the target orfs were first PCR amplified with Phusion High-Fidelity polymerase (Thermo Fisher) for both the *S. pneumoniae* strains Tigr4 or D39 (the same strain background was always used as the template for the PCR as becoming the host for the transformation). The primers used are listed in Table S1 in which the target gene-specific UpFr and UpRe primers and DoFr and DoRe were used to amplify the up- and down-stream regions, respectively. The respective UpRe and DoFr primers contained a non-annealing overhang sequence complementary to the antibiotic resistance Orf (or cassette) to be fused **Table S1** and **Fig. S2A**. These antibiotic resistance Orfs (or cassettes) were also PCR amplified with specific primers given in **Table S1**. The PCR products were analyzed with agarose gel electrophoresis and purified with QIAquick PCR Purification Kit (Qiagen). In the following step (**Fig. S2B**), the up- and the down-region amplicons were individually fused to the antibiotic Orf/cassette amplicon through thermal annealing and elongation for 6 cycles (without primers) to join and extend both strands followed by PCR amplification in presence of primers binding to the 5' and 3' ends of the fused template. In the final step, the (up-region - antibiotic Orf/cassette) were fused to the (antibiotic Orf/cassette - down-region) by iterating the procedure (6 cycles of thermal annealing and elongation followed by PCR amplification) using the gene specific UpFr and DoRe primers (**Table S2** and **Fig. S2C**). The final PCR products that contained the joined up-stream region fused to an antibiotic Orf (or cassette) fused to the down-stream region and these fragments were purified (QIAquick PCR Purification Kit Qiagen) and used for pneumococcal transformation. Pneumococcal transformation was performed as described previously (1) using competence stimulating peptide 1 for transforming strains in a D39 background. Transformants were selected on blood agar plates containing 2 mg/L erythromycin, 200 mg/L kanamycin and 200 mg/L spectinomycin. Using this methodology to construct transformation amplicons we generated the following mutant isolates: D39Δ*fakB3* (BHN2024), BHN857Δ*fakB3* (BHN2025), D39Δ*fakB1* (BHN2030), D39Δ*fakB2* (BHN2031), D39Δ*fakB1*Δ*fakB2* (BHN1351), D39Δ*fabT* (BHN2032), D39Δ*lytA* (BHN2042), T4Δ*fakB3* (BHN2033), T4Δ*fabT* (BHN2034), T4Δ*SP0743* (BHN2037), T4Δ*SP0741* (BHN2038), T4Δ*0740* (BHN2039), T4Δ*SP0740-SP0742* (BHN2040) (**Table S1, Fig. S3 and S4**).

A similar approach was used to generate strains in which mutant alleles of *fabT* were replaced with the wildtype *fabT* allele. To facilitate cloning and selection of transformants, an *ermB* Orf was inserted directly behind the *fabT* stop codon maintaining the same intertwined start/stop codon context as shared with the *fabT* and *fabH* Orfs (see **Fig. S3**). Thus, a transformation construct was made using the same sequential extension PCR methodology as described above but instead of replacing a target gene the two adjacent regions flanking the *fabT* stop codon were PCR amplified in the first step (**Fig. S2A**) and used to join in an *ermB* orf with the same approach as in the subsequent steps in the procedure described (**Fig. S2**). Primers used were fabT-UpFr and fabTOrf-UpRe-ermOrf-OH that amplified an 'up-region' including the whole *fabT* Orf plus a 600 bp region upstream of *fabT*, and primers fabT-DoFr-ermOrf-OH plus fabT-DoRe were used to amplifly the 600 bp downstream of the *fabT* stop codon. These fragments were amplified from the wildtype Tigr4 and D39 genomic DNA to contain the wildtype *fabT* allele. The subsequent transformation constructs made were used to transform the *fabT* mutant strains BHN848 and BHN859 to replace their mutant alleles with wildtype version. Following transformation, strains BHN2036 and BHN2023 were isolated and Sanger sequencing confirmed that they contained the wildtype *fabT* allele plus the inserted *ermB* Orf. In parallel, also the Tigr4 and D39 wildtype strains (BHN842 and BHN853) were transformed with the same transformation constructs yielding strains BHN2035 and BHN2022 that served as control

strains with the same *ermB* Orf insertion into the *fabT* operon (see **Fig. S3** for an overview of the mutant strains in the *fabT* gene context).
The same methodology was used to replace the a *fakB3* mutant allele in strain BHN857 with the wildtype *fakB3* allele to generate BHN2028. The same construct was used to transform the D39 wildtype strain to generate BHN2027, which served as a control strain having an *ermB* cassette inserteded downstream the *fakB3* locus. The transformation PCR amplicon contained an 'up-region' including the wildtype *fakB3* Orf plus a 600 bp region upstream *fakB3* start codon, joined to an *ermB* cassette which was fused to a 600 bp region downstream the *fakB3* stop codon. See **Fig. S4** for an overview of the *fakB3* mutant strains and mutants in the neighboring Orfs.

2CCA-1 sensitivity testing of *Staphylococcus aureus*

S. aureus (ATCC 25923) was cultivated overnight on blood agar plates. Suspension cultures were grown in BHI medium supplemented with horse serum (1 % (v/v), Håtunalab) and glucose bouillon (9 % (v/v), 25 g/L nutrient broth No.2 (Oxoid); 10 g/L glucose). A pre-culture of *S. aureus* was grown at 37 ºC with shanking at ~200 rpm until mid-log phase and subsequently diluted to $OD_{600nm} \approx 0.05$ into fresh pre-warmed supplemented BHI medium. Thereafter 2CCA-1 sensitivity testing was performed as described in the assay for assessing the lysis inducing activity on *S. pneumoniae*.

Lipid extraction

Bacterial pellets were thawed on ice and 500 µL of extraction mix (chloroform: methanol 2:1 (v:v) including internal standards) was added to the tube with the bacterial cells. Two tungsten beads were added, and the samples were shaken in a bead mill (3 min at 30Hz). The samples were allowed to stand on ice for 2 h. After this, 0.9 % NaCl (100 µL) was added and all samples were centrifuged at 14000 rpm for 3 min. The lower phase (300 µL) was transferred to a LC vial and dried to complete evaporation in a vacuum chamber (15-20 min). After this, extracted lipids were dissolved in 30 µL chloroform and kept at -80 ˚C until the analysis.

Liquid chromatography - mass spectrometry

Total lipids were analyzed by Liquid chromatography - mass spectrometry (LC-MS) using an Infinity 1290 Agilent (Agilent Technologies, Santa Clara, CA, USA) ultra-high-performance liquid chromatograph coupled with tandem mass spectrometry (UHPLC-MS-MS). The UHPLC system included a precolumn (2.1 mm × 5 mm, 1.7 µm VanGuard CSH; Waters Corporation, Milford, MA, USA) followed by a column (CSH, 2.1 × 50 mm, 1.7 µm C18, Acquity) as previously described (5). The system was kept at 60 ºC and for analysis, 1 µL of a sample was injected per run. The gradient elution buffers were: A (60:40 acetonitrile: water, 10 mM ammonium formate containing 0.1 % formic acid) and B (90:10 2-propanol: acetonitrile, 10 mM ammonium formate containing 0.1 % formic acid). The run started at 15 % B buffer, flow rate 0.5 mL/min. Further, B was increased to 30 % in 1.2 min, then to 55 % in 0.3 min, and held at 55 % for 3.5 min after which it was progressively increased as follows: 72 % in 2 min, then 85 % in 2.5 min, and to 100 % in 0.5 min and was held for 2 min.
Lipid masses were detected in positive and in negative mode using an Agilent 6550 Q-TOF mass spectrometer and an iFunnel jet stream electrospray ion source (Agilent Technologies, Santa Clara, CA, USA). The flow gas temperature was 150 °C, the drying gas flow was 12 L/min, and the nebulizer pressure at 40 psi. The sheath gas temperature was 350 °C and the sheath gas flow was 1 L/min. The capillary voltage was 4000 V for the positive mode and 2300 V for the negative mode. The m/z range was 70–1700, and data were collected in centroid mode; acquisition rate of 4 scans per second. Selected lipid compounds were characterized by targeted MS/MS method. Spectra were processed using MassHunter ProFinder B.10.00 (Agilent) with standard settings.

Protein expression and purification

Recombinant FabT was produced in BL21 cells (One shot BL21 Star (DE3) (Thermo Fisher Scientific)) containing the pET21d-fabT-6His expression vector. Cells were grown in a 50:50 (%) mixture of Luria broth and Terrific broth (both from Merck) containing ampicillin (100 mg/L) at 37 ºC with agitation. Protein expression was induced at an OD_{600nm} of 0.5 with 1 mM isopropyl 1-thio-β-d-galactopyranoside (IPTG) and cells were harvested by centrifugation following 4 hours of growth and cell pellets were frozen (-20 ºC). Cell pellets were thawed and resuspended in lysis buffer (20 mM Tris, (pH 7.5), 500 mM NaCl, 5 mM imidazole with lysozyme (1 mg/mL), DNase I (200 µg/mL) and 1 x Complete Protease Inhibitor Cocktail) and incubated at 4 ºC for 30 min with agitation. The cells were broken by three passages through the chamber of an Ultra High-Pressure Homogenizer (Homogenising Systems Ltd) operating at 2000 psi and the resulting cell debris was removed by centrifugation. The clarified cell lysate was applied to a gravity flow column containing Ni Sepharose 6 Fast Flow (GE Healthcare) equilibrated with binding buffer (20 mM Tris pH 7.5, 500 mM NaCl, 5 mM imidazole). After passage of the cell lysate the column were washed with 20 column volumes of wash buffer (20 mM Tris pH 7.5, 500 mM NaCl, 60 mM imidazole) and elution was done with elution buffer (20 mM Tris pH 7.5, 500 mM NaCl, 300 mM imidazole). Protein-containing fractions were pooled and dialyzed against buffer (150 mM KCl, 10 mM Tris, 0.1 mM DTT and 0.1 mM EDTA) (6). Protein concentration was determined by reading the absorbance at 280 nm using the molar extinction coefficient 5960 cm/M and the molecular mass 17558 Da.

Electromobility shift assay

Recombinant FabT was produced and purified as described above. To facilitate the migration of the FabT:DNA complex to the anode during electrophoresis, 300 bp DNA fragments were used to provide a net-negative charge of the complex (7). A 300 bp fragment of the *fakB3* promoter was produced as described above. The FabT binding sites in the *fabT* promoter (5'-AATGTTTTGATTGTAAAAGTTTT-3') and *fabK* promoter (5'-AATAGTTTGACTGTCAAATTATG-3') (7-9) were embedded in a 300 bp DNA fragment with flanking sequences of the pneumococcal GAPDH Orf (SPD_1823). A similar 300 bp sequence of the GAPDH Orf, but with no FabT binding site, was used as unspecific competitor. The fragments were synthesized as dsDNA (Eurofins Genomics). All investigated 300 bp fragments were amplified using primers given in Table S2 and purified from 1% agarose gels using the QIAquick PCR purification kit (Qiagen). DNA (210 ng) was radioactively end-labelled for 30 min at 37 ºC using T4 Polynukleotide Kinase (0.33 units/uL, New England Biolabs), [γ-^{32}P]ATP (0.17 µCi/µL, Perkin Elmer) and T4 PNK reaction buffer (1x, New England Biolabs) in a volume of 30 µL. Reactions were purified using llustra™ MicroSpin G-25 columns (GE Healthcare) according to the manufacturer's instructions and the elution volume was adjusted with water to 40 µL for all samples. DNA concentration after purification was calculated accounting a yield of 80 % for column purification and repeated labelling and purification was performed for all probes. For the binding reactions all components were diluted in binding buffer (Tris (20 mM, pH 7.3), NaCl (50 mM), $MgCl_2$ (10 mM), $CaCl_2$ (5 mM), BSA (0.2 mg/mL), Glycerol (10 %), DTT (1 mM) and EDTA (1 mM)) and each reaction was adjusted to 10 µL with the binding buffer. For competition reactions, 10 to 40-fold of the respective unlabeled fragment and the same excess of 300 bp GAPDH fragment containing no binding site were added before the labelled DNA (0.4 nM). After 20 min incubation at room temperature, and addition of 1 µL loading dye (60 % (m/v) sucrose, 0.25 % (m/v) bromothymol blue and 0.25 % (m/v) xylene cyanol FF), electrophoresis was performed in agarose gels (0.5 % m/v) dissolved in TBE running buffer (0.5 x, Sigma) for an initial 5 min at 110 V and 20 min at 90 V. Gels were dried (Phero-Temp 60, Biotec Fisher) before autoradiography.

Membrane fluidity determination:

2CCA-1 induced changes in pneumococcal membrane fluidity were assessed by determining laurdan general polarization, with a protocol adapted from previously published work (10-12). Liquid cultures of wildtype D39 were prepared as in the assays for lysis inducing activity. At OD 0.15 cultures were treated with 3 µM (1 x MlytC), 25 µM (8 x MlytC), 50 µM (16 x MlytC) and 100 µM (32 x MlytC) 2CCA-1 as well as 1 % (v/v) DMSO as solvent control. Treatment with 30 mM Benzylalcohol (Sigma) was included as a positive control for membrane fluidization (13). At OD ~0.23, 400 µL of bacterial culture were stained with 10 µM laurdan (6-Dodecanoyl-*N*,*N*-dimethyl-2-naphthylamine, Sigma) dissolved in dimethyl formamide to a final solvent concentration of 0.1% for 5 min at room temperature. Thereafter, cells were washed four times with pre-warmed PBS+2 % (m/v) glucose with two-minute centrifugation steps at 6200xg. The pellet was taken up in 200 µL pre-warmed PBS+ 2 % glucose and transferred to a pre-warmed black microwell plate for immunoanalytics (Sarstedt). Fluorescent intensities were recorded at 37 °C in the SpextraMax plate reader (Molecular devices) and emission wavelengths of 460 ± 15 nm and 500 ± 15 nm upon excitation with 350 ± 9 nm. Laurdan general polarization GP = $(I_{460} – I_{500}) / (I_{460} + I_{500})$ was calculated. Membrane fluidity determination was performed in at least technical duplicates and biological triplicates.

Supplementary references:

1. Mellroth P, Daniels R, Eberhardt A, Rönnlund D, Blom H, Widengren J, Normark S, Henriques-Normark B. 2012. LytA, major autolysin of Streptococcus pneumoniae, requires access to nascent peptidoglycan. Journal of Biological Chemistry 287:11018-11029.
2. Macrina FL, Evans RP, Tobian JA, Hartley DL, Clewell DB, Jones KR. 1983. Novel shuttle plasmid vehicles for Escherichia-Streptococcus transgeneric cloning. Gene 25:145-50.
3. Bubeck P, Winkler M, Bautsch W. 1993. Rapid cloning by homologous recombination in vivo. Nucleic Acids Res 21:3601-2.
4. Jones DH, Howard BH. 1991. A rapid method for recombination and site-specific mutagenesis by placing homologous ends on DNA using polymerase chain reaction. Biotechniques 10:62-6.
5. Diab J, Hansen T, Goll R, Stenlund H, Ahnlund M, Jensen E, Moritz T, Florholmen J, Forsdahl G. 2019. Lipidomics in Ulcerative Colitis Reveal Alteration in Mucosal Lipid Composition Associated With the Disease State. Inflamm Bowel Dis 25:1780-1787.
6. Jing D, Agnew J, Patton WF, Hendrickson J, Beechem JM. 2003. A sensitive two-color electrophoretic mobility shift assay for detecting both nucleic acids and protein in gels. Proteomics 3:1172-80.
7. Jerga A, Rock CO. 2009. Acyl-Acyl carrier protein regulates transcription of fatty acid biosynthetic genes via the FabT repressor in Streptococcus pneumoniae. J Biol Chem 284:15364-8.
8. Lu YJ, Rock CO. 2006. Transcriptional regulation of fatty acid biosynthesis in Streptococcus pneumoniae. Mol Microbiol 59:551-66.
9. Zuo G, Chen ZP, Jiang YL, Zhu Z, Ding C, Zhang Z, Chen Y, Zhou CZ, Li Q. 2019. Structural insights into repression of the Pneumococcal fatty acid synthesis pathway by repressor FabT and co-repressor acyl-ACP. FEBS Lett 593:2730-2741.
10. Saeloh D, Tipmanee V, Jim KK, Dekker MP, Bitter W, Voravuthikunchai SP, Wenzel M, Hamoen LW. 2018. The novel antibiotic rhodomyrtone traps membrane proteins in vesicles with increased fluidity. PLoS Pathog 14:e1006876.
11. Wenzel M, Vischer NOE, Strahl H, Hamoen LW. 2018. Assessing Membrane Fluidity and Visualizing Fluid Membrane Domains in Bacteria Using Fluorescent Membrane Dyes. Bio-protocol 8:e3063.
12. Müller A, Wenzel M, Strahl H, Grein F, Saaki TNV, Kohl B, Siersma T, Bandow JE, Sahl HG, Schneider T, Hamoen LW. 2016. Daptomycin inhibits cell envelope synthesis by interfering with fluid membrane microdomains. Proc Natl Acad Sci U S A 113:E7077-e7086.
13. Friedlander G, Le Grimellec C, Giocondi MC, Amiel C. 1987. Benzyl alcohol increases membrane fluidity and modulates cyclic AMP synthesis in intact renal epithelial cells. Biochim Biophys Acta 903:341-8.
