# Supplementary material for: The Bactericidal Fatty Acid Mimetic 2CCA-1 Selectively Targets Pneumococcal Extracellular Polyunsaturated Fatty Acid Metabolism
Source: mBio. 2020 Dec 15;11(6):e03027-20. doi: 10.1128/mBio.03027-20 (PMC7773995; doi:10.1128/mBio.03027-20)
Supplement: TABLE S1 [file mBio.03027-20-st001.pdf]

Table S1. Pneumococcal strains used in the study

Strain	Identifyer	Genotype	Reference	WGS	Fig.[a]
D39	BHN853	D39, wildtype, Serotype 2, (parental strain for BHN857-BHN860 and BHN870)	Laboratory collection	WGS	
D39-*fakB3*-mutant_1	BHN857	Spontaneous 2CCA-1 resistant D39 strain isolated after 2 hours exposure to 2CCA-1, includes a mutation in *fakB3*	This study	WGS	S3
D39-*fakB3*-mutant_2	BHN858	Spontaneous 2CCA-1 resistant D39 strain isolated after 2 hours exposure to 2CCA-1, includes a mutation in *fakB3*	This study	WGS	
D39-*fabT*-mutant_1	BHN859	Spontaneous 2CCA-1 resistant D39 strain isolated after 2 hours exposure to 2CCA-1, includes a mutation in *fabT*	This study	WGS	S2
D39-*fakB3*-mutant_3	BHN860	Spontaneous 2CCA-1 resistant D39 strain isolated after overnight exposure to 2CCA-1, includes a mutation in *fakB3*	This study	WGS	S3
D39-*fakB3*-mutant_4	BHN870	Spontaneous 2CCA-1 resistant D39 strain isolated after overnight exposure to 2CCA-1, includes a mutation in *fakB3*	This study	WGS	
D39Δ*fakB3::ermB*	BHN2024	Strain BHN853 with Orf *SPD0646* encoding "FakB3" replaced with an *ermB* cassette	This study		S3
BHN857Δ*fakB3::ermB*	BHN2025	Strain BHN857 with Orf *SPD0646* encoding "FakB3" replaced with an *ermB* cassette	This study		S3
D39(*SPD0645*-∇*ermB*-*SP0646*)	BHN2027	Strain BHN853 with *ermB* cassette inserted between *fakB3* (*SPD0646*) and (*SPD0645*)	This study		S3
BHN857∇*fakB3*-(*SPD0645*-∇*ermB*-*SP0646*)	BHN2028	Mutant *fakB3* allele of BHN857 replaced with a wildtype *fakB3* allele and with an *ermB* cassette inserted between *fakB3* (*SPD0646*) and (*SPD0645*)	This study		S3
D39Δ*fakB1*::*kanR*	BHN2030	*fakB1* (*SPD1388)* Orf replaced with a kanamycin resistance cassette	This study		
D39Δ*fakB2*::*spcR*	BHN2031	*fakB2* (*SPD0996)* Orf replaced with a spectinomycin resistance cassette	This study		
D39Δ*fakB1::ermB*Δ*fakB2::tetR*	BHN1351	*fakB1* (*SPD1388)* Orf replaced with an erythromycin resistance Orf; *fakB2* (*SPD0996)* Orf replaced with a tetracyclin resistance Orf	This study		
D39Δ*fabT::ermB*	BHN2032	Orf *SPD0379* encoding "FabT" replaced with an *ermB* Orf	This study		S2
D39(*SPD0379*-∇*ermB*- *SPD0380*)	BHN2022	*ermB* Orf inserted between *fabT* (*SPD0379*) and *fabH* (*SPD0380*)	This study		S2
BHN859∇*fabT*-(*SPD0379*-∇*ermB*- *SPD0380*)	BHN2023	Mutant *fabT* allele of BHN859 replaced with a wildtype *fabT* allele and with an *ermB* Orf inserted between *fabT* (*SPD0379*) and *fabH* (*SPD0380*)	This study		S2
D39Δ*lytA::ermB*	BHN2042	Orf *SPD1737* encoding "LytA" replaced with an *ermB* cassette	This study		

Strain	Identifyer	Genotype	Reference	WGS	Fig.[a]
Tigr4 (T4)	BHN842	Tigr4, wildtype, Serotype 4 (parental strain for BHN848-BHN852)	Laboratory collection	WGS	
T4-*fabT*-mutant_1	BHN848	Spontaneous 2CCA-1 resistant Tigr4 strain isolated after 2 hours exposure to 2CCA-1, includes a mutation in *fabT*	This study	WGS	S2
T4-*fakB3*-mutant_1	BHN849	Spontaneous 2CCA-1 resistant Tigr4 strain isolated after 2 hours exposure to 2CCA-1, includes a mutation in *fakB3*	This study	WGS	
T4-*fakB3*-mutant_2	BHN850	Spontaneous 2CCA-1 resistant Tigr4 strain isolated after 2 hours exposure to 2CCA-1, includes a mutation in *fakB3*	This study	WGS	
T4-*fakB3*-mutant_3	BHN851	Spontaneous 2CCA-1 resistant Tigr4 strain isolated after overnight exposure to 2CCA-1, includes a mutation in *fakB3*	This study	WGS	
T4-*fakB3*-mutant_4	BHN852	Spontaneous 2CCA-1 resistant Tigr4 strain isolated after overnight exposure to 2CCA-1, includes a mutation in *fakB3*	This study	WGS	
T4Δ*fakB3::ermB*	BHN2033	Orf SP0742 encoding “FakB3” replaced with an *ermB* cassette	This study		S3
T4Δ*fabT::ermB*	BHN2034	Orf SP0416 encoding “FabT” replaced with an *ermB* Orf	This study		S2
T4(*SP0416*-∇*ermB*-*SP0417*)	BHN2035	*ermB* Orf inserted between *fabT* (*SP0416*) and *fabH* (*SP0417*)	This study		S2
BHN848∇*fabT*-(*SP0416*-∇*ermB*-*SP0417*)	BHN2036	Mutant *fabT* allele of BHN848 replaced with a wildtype *fabT* allele and with an *ermB* Orf inserted between *fabT* (*SP0416*) and *fabH* (*SP0417*)	This study		S2
T4Δ*SP0743::ermB*	BHN2037	Orf *SP0743* encoding “transcriptional regulator, TetR family”, replaced with an *ermB* cassette	This study		S3
T4Δ*SP0741::ermB*	BHN2038	Orf *SP0741* encodes “NAD(P)/FAD-dependent oxidoreductase”, replaced with an *ermB* cassette	This study		S3
T4Δ*SP0740::ermB*	BHN2039	Orf *SP0740* encodes “MutT/NUDIX family protein”, replaced with an *ermB* cassette	This study		S3
T4Δ*SP0740-SP0742::ermB*	BHN2040	Orfs *SP0740-SP0742* replaced with an *ermB* cassette	This study		S3

Notes: [a]) See indicated Supplementary figure for extended information about the strain.
