# Supplementary material for: The Bactericidal Fatty Acid Mimetic 2CCA-1 Selectively Targets Pneumococcal Extracellular Polyunsaturated Fatty Acid Metabolism
Source: mBio. 2020 Dec 15;11(6):e03027-20. doi: 10.1128/mBio.03027-20 (PMC7773995; doi:10.1128/mBio.03027-20)
Supplement: TABLE S2 [file mBio.03027-20-st002.pdf]

Table S2. Primers used in the study

Primer	Sequence (5'-3')[a]	Comment
Primers for making transformation constructs		
lytA-UpFr	tgacctttgcccttcttcct	
lytA-UpRe-ermCassOH	TGCAAGTCACACGAACACGAAattctactccttatcaattaaaac	Non-annealing overhang (OH) seq complimentary to 5'-end of *ermB* cassette
lytA-DoFr-ermCassOH	CTCGTCTTAATCAGCAACATtaatggaatgtctttcaaatc	OH seq complimentary to 3'-end of *ermB* cassette
lytA-DoRe	tctctgatttcctcaaccatc	
fakB3-UpFr	tctcttttctccttttctct	
fakB3-UpRe-ermCassOH	GCAAGTCACACGAACACGAAagaaattctccatctttgtcag	OH seq complimentary to 5'-end of *ermB* cassette
fakB3-DoFr-ermCassOH	CTCGTCTTAATCAGCAACATttgcattcttgacaagaggt	OH seq complimentary to 3'-end of *ermB* cassette
fakB3-DoRe	tagggtcacatcgtccagaga	
fakB3-Orf-UpRe-ermCassOH	TGCAAGTCACACGAACACGAAttaatcaatttcatagccca	OH seq complimentary to 5'-end of *ermB* cassette
fakB1-UpFr	ccacaaaaattagttaatatcc	
fakB1-UpRev-kanCassOH	TGCAAGTCACACGAACACGAAattagaactccaaattaattcct	OH seq complimentary to 5'-end of *kan* cassette
fakB1-DoFrw-kanCassOH	TGCTCGTCTTAATCAGCAACATttggcgcttctaggctagttaag	OH seq complimentary to 3'-end of *kan* cassette
fakB1-UpRe-ermOrfOH	GAGAATATTTTATATTTTTGTTCATattagaactccaaattaattc	OH seq complimentary to 5'-end of *ermB* Orf
fakB1-DoFr-ermOrfOH	CTATTATTTAACGGGAGGAAATAAttggcgcttctaggctagttaag	OH seq complimentary to 3'-end of *ermB* Orf
fakB1-DoRe	tccacatttgggaaatatttg	
fakB2-UpFr	aacacgagaaacaataaaatc	
fakB2-UpRe-spcCassOH	TGCAAGTCACACGAACACGAAagtatcaatctttctattttagg	OH seq complimentary to 5'-end of *spcR* cassette
fakB2-DoFr-spcCassOH	TGCTCGTCTTAATCAGCAACATaaaataaataaaattggaagaaat	OH seq complimentary to 3'-end of *spcR* cassette
fakB2-DoRe	cttcactatcttgttggattttc	
fakB2-UpRe-tetOrfOH	TTTTCATGTGATTTTCCTCCATagtatcaatctttctattttagg	OH seq complimentary to 5'-end of *tetM* Orf
fakB2-DoFr-tetOrfOH	TGTTCAATAAAATAACTTAGaaaataaataaaattggaagaaatagc	OH seq complimentary to 3'-end of *tetM* Orf
fabT-UpFr	tcaagcaaatagccaaacct	
fabT-UpRe-ermOrf-OH	GAGAATATTTTATATTTTTGTTCATttttcatatccctccttctt	OH seq complimentary to 5'-end of *ermB* Orf
fabT-DoFr-ermOrf-OH	CTATTATTTAACGGGAGGAAATAAtggcttttgcaaaaataagtcag	OH seq complimentary to 3'-end of *ermB* Orf
fabT-DoRe	ctgaaaatggagaatgcaaac	

Primer	Sequence (5'-3')[a]	Comment
fabTOrf-UpRe-ermOrf-OH	TGAGAATATTTTATATTTTTGTTCAttatttcaaatcctccaaaaattgg	OH seq complimentary to 5'-end of *ermB* Orf
SP0743-UpFr	caacttcaccaccagcagat	
SP0743-UpRe-ErmCass-OH	GCAAGTCACACGAACACGAAaacatttctttctacttgtca	OH seq complimentary to 5'-end of *ermB* cassette
SP0743-DoFr-ErmCass-OH	CTCGTCTTAATCAGCAACATagaaaaaggagaaaagagatga	OH seq complimentary to 3'-end of *ermB* cassette
SP0743-DoRe	gatcagcctctttaagagct	
SP0741-UpFr	tgtcttttgaagaagtggtt	
SP0741-UpRe-ermCass-OH	TCGTCTTAATCAGCAACATagctctattgtaccacaa	OH seq complimentary to 5'-end of *ermB* cassette
SP0741-DoFr-ermCass-OH	AGTCACACGAACACGAAagattaaaggttgtgaaga	OH seq complimentary to 3'-end of *ermB* cassette
SP0741-DoRe	atgcagaataaataggcta	
SP0740-UpFr	actttggtttgtcaggtcct	
SP0740-UpRe-ermCass-OH	GCTCGTCTTAATCAGCAACATtcttcacaacctttaatcttta	OH seq complimentary to 5'-end of *ermB* cassette
SP0740-DoFr-ermCass-OH	GCAAGTCACACGAACACGAAaaaataggctaggaaattccta	OH seq complimentary to 3'-end of *ermB* cassette
SP0740-DoRe	acatctggataccttgatttc	
Primers used to construct antibiotic resistance cassettes		
pSp72-Fr	ttctatgagtcgcttttgtaaat	
pSp72-Re	gtaatcactccttcttaattac	
kanOrf-Fr-pSp72-OH	GTAATTAAGAAGGAGTGATTACatggctaaaatgagaatatca	
kanOrf-Re-pSp72-OH	ATTTACAAAAGCGACTCATAGAActaaaacaattcatccagtaa	
spcOrf-Fr-pSp72-OH	GTAATTAAGAAGGAGTGATTACatgaatacatacgaacaaatta	
spectOrf-Re-pSp72-OH	ATTTACAAAAGCGACTCATAGAActataattttttaatctgttattta	
Primers used to amplify antibiotic resistance Orfs or cassettes		
AbCass-Fr	ttcgtgttcgtgtgacttgca	
AbCass-Re	atgttgctgattaagacgagca	
ErmOrf-Fr	atgaacaaaaatataaaatattctc	
ErmOrf-Re	ttatttcctcccgttaaataatag	
KanOrf-Fr	atggctaaaatgagaatatca	
KanOrf-Re	ctaaaacaattcatccagtaaaa	
TetOrf-Fr	atggaggaaaatcacatgaaaa	
TetOrf-Re	ctaagttattttattgaacatatatc	

Primer	Sequence (5'-3')[a]	Comment
Primers used to produce amplicons for sequencing of transformants		
lytUpFr-seq	atgagttcaattgtatctatc	
lytDoRe-seq	cattcctcaatctatataaca	
fakB3-UpFr-seq	gaatcgcgcccactttagcac	
fakB3-DoRe-seq	ctttgacaaagctagacatgc	
fakB1-UpFr-seq	tacaataccacaggtgatggt	
fakB1-DoRe-seq	cttgctgaatggactctcga	
fakB2-UpFr-seq	cttccgaggtcatcattaatc	
fakB2-DoRe-seq	agtatagcatgtttccctaag	
fabT-UpFr-seq	gagatttggtagagatgaag	
fabT-DoRe-seq	tcaatagtctgcttgatagact	
tesS-UpFr-seq	ctgaaactggctggttttgaca	
tesS-DoRe-seq	gtaaaacctgtcgtcaccaag	
acpB-UpFr-seq	acgagagctattcggaataag	
acpB-DoRe-seq	aatccataattaccaccaaca	
SP0743-UpFr-seq	tttttcttgataggcggtga	
SP0743-DoRe-seq	ccatttttggaattccttct	
SP0741-UpFr-seq	atatttatctggaagaccat	
SP0741-DoRe-seq	agagtttaaggaaaacattga	
SP0740-UpFr-seq	agattttccacataaagcct	
SP0740-DoRe-seq	atagcagagctgttaaagga	
Primers for cloning fakB3 promoter into pPP2 vector		
pPP2-Fr	atgaaacatcttgatcccgtc	Forward primer for amplifying pPP2
pPP2-Re	gattctagactagaattcgca	Reverse primer for amplifying pPP2
PfakB3-Fr-pPP2-OH	TGCGAATTCTAGTCTAGAATCaacattttctttctacttgt	Amplifies fakB3 promotor with an overhang sequence complementary to 3' end of the PCR amplified pPP2 vector
PfakB3-Re-pPP2-OH	ACGGGATCAAGATGTTTCATagaaattctccatctttgtca	Amplifies fakB3 promotor with an overhang sequence complementary to 5' end of the PCR amplified pPP2 vector
pPP2-seq-Fr	tgctcgcttcgctacttgga	Forward primer – amplifies a 308 bp fragment including the *fakB3* promoter
pPP2-seq-Re	aggcgattaagttgggtaac	Reverse primer – amplifies a 308 bp fragment including the *fakB3* promoter

Primer	Sequence (5'-3')[a]	Comment
Primers for cloning fabT-c-6His into pET21d protein expression vector		
pET21-Fr-6His-OH	CACCATCACCATCACCATTAAaccaagcttgggcccgaaca	Forward primer for amplifying pET21d with an overhang sequence including six histidine codons plus a TAA Stop codon
pET21-Re	gctagccaaggtatatctcct	Reverse primer for amplifying pET21d
fabT-Fr-pET21-OH	GGAGATATACCTTGGCTAGCatggactaccaacgaatcaat	Forward primer for amplifying *fabT* Orf with an overhang sequence complementary to the 3'end of the amplified pET21 vector
fabT-Re-6His-OH	TTAATGGTGATGGTGATGGTGtttcaaatcctccaaaaattggt	Forward primer for amplifying *fabT* Orf with an overhang sequence including six histidine codons plus a TAA Stop codon complementary to the 5'end of the amplified pET21 vector
Primers for amplifying the 300bp GAPDH fragments used for EMSA experiments		
GAPDH300-Fr	aaagttggtattaacggtttcg	Forward primer for amplifying the 300bp GAPDH fragments with FabT binding sites from the *fabT* and *fabK* promoter included, and with no binding site
GAPDH300-Re	tttcttagcaaagaaaccagtagc	Reverse primer for amplifying the 300bp GAPDH fragments with FabT binding sites from the *fabT* and *fabK* promoter included, and with no binding site
qPCR primer		
fakB3-qPCR-Fr	caactgccaacaccagctat	
fakB3-qPCR-Rev	ccttcaaatgctcgcaaata	
fabK-qPCR-Fr	ccctttgtggaagacatcgt	
fabK-qPCR-Rev	cgattatcccagcttcatgg	
gyrA-qPCR-Fr	gttcgcttggttcaggaaaa	
gyrA-qPCR-Rev	ttgcatttgggtcattttga	

Notes: [a]) Annealing sequences are displayed in lower case letters and non-annealing overhang sequences in upper case letters
