# Supplementary material for: The Bactericidal Fatty Acid Mimetic 2CCA-1 Selectively Targets Pneumococcal Extracellular Polyunsaturated Fatty Acid Metabolism
Source: mBio. 2020 Dec 15;11(6):e03027-20. doi: 10.1128/mBio.03027-20 (PMC7773995; doi:10.1128/mBio.03027-20)
Supplement: FIG S2 [file mBio.03027-20-sf002.pdf]

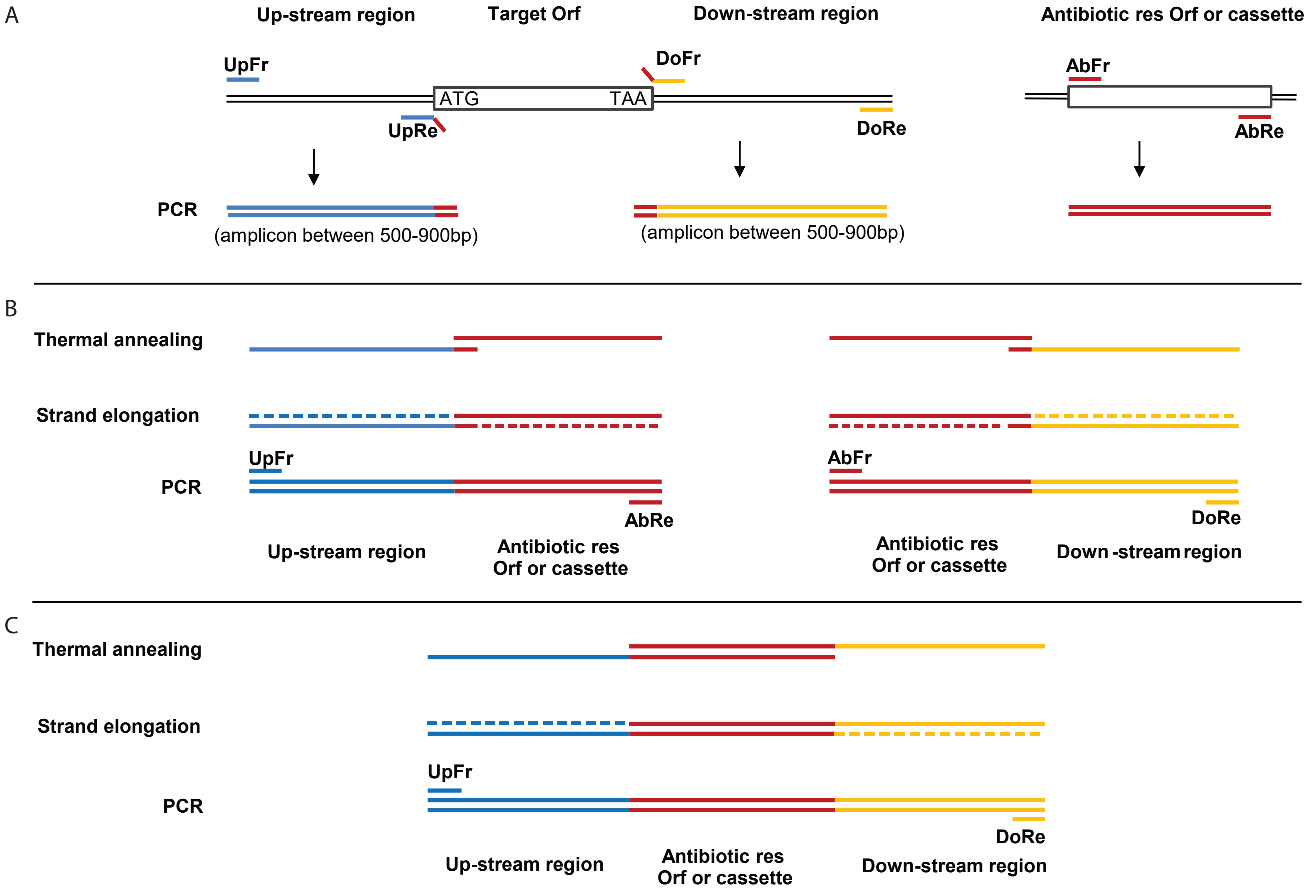

Figure S2. Sequential overlap PCR methodology for making transformation constructs.
(A) The up- and down-stream regions of target genes were PCR amplified to produce amplicons that covered regions (of 500 to 900bp). The primers that flanked the target gene contained non-annealing overhang sequences (depicted in red) complimentary to the 5' or 3' ends of an antibiotic resistance Orf/cassette. Also, the corresponding antibiotic resistance Orf/cassette was amplified with specific primers. **(B)** In the next step the up-region and the down-region were separately joined to the antibiotic resistance Orf/cassette through thermal annealing followed by strand elongation (without primers) for six cycles and then PCR amplification was performed with primers that annealed to the 5' and 3' ends of the amplicon. **(C)** Finally, the two PCR products produced in (B) were joined by thermal annealing-elongation and PCR as described in (B).
