# Supplementary material for: The Bactericidal Fatty Acid Mimetic 2CCA-1 Selectively Targets Pneumococcal Extracellular Polyunsaturated Fatty Acid Metabolism
Source: mBio. 2020 Dec 15;11(6):e03027-20. doi: 10.1128/mBio.03027-20 (PMC7773995; doi:10.1128/mBio.03027-20)
Supplement: FIG S3 [file mBio.03027-20-sf003.pdf]

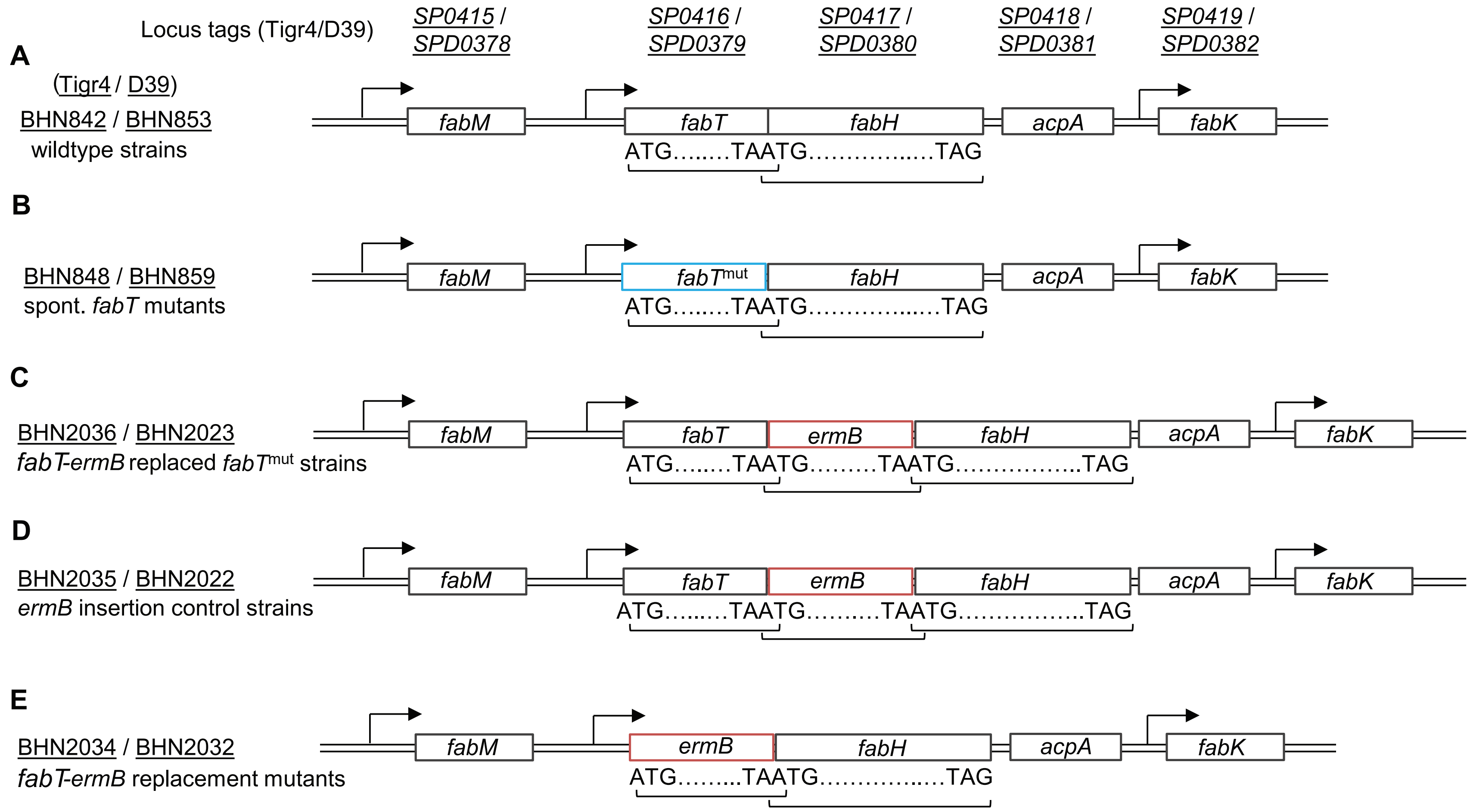

Figure S3. Overview of the *fabT* operon in the wildtype and mutant strains produced in the study.
(A) An illustration of the gene segment covering Orfs *SP0415-19* (Tigr4) and *SPD0378-82* (D39) including the *fabT* operon and the flanking *fabM* and *fabK* Orfs. Location of promotors are indicated with arrows displaying the direction of transcription. The TAA stop codon of *fabT* is intertwined with the ATG start codon of *fabH*. **(B)** Strains BHN848 and BHN859 (Tigr4 and D39, respectively) contain a spontaneous *fabT* mutation selected after 2A treatment. **(C)** Strains (BHN2036 and BHN2023) are derivatives of the spontaneous *fabT* mutant strains BHN848 and BHN859 where the *fabT* allele was replaced with a wildtype *fabT* allele. An *ermB* Orf was inserted between *fabT* and *fabH* Orfs maintaining a similar intertwined Stop/Start-codon context. **(D)** Control strains (BHN2035 and BHN2022) in which an *ermB* Orf was inserted between *fabT* and *fabH* Orfs in the wildtype Tigr4 and D39 (BHN842 and BHN853) backgrounds. **(E)** Mutants (BHN2034 and BHN2032) in which the *fabT* Orf was replaced with an *ermB* Orf with maintained intertwined Stop/Start-codon with *fabH*.
