# Supplementary material for: The Bactericidal Fatty Acid Mimetic 2CCA-1 Selectively Targets Pneumococcal Extracellular Polyunsaturated Fatty Acid Metabolism
Source: mBio. 2020 Dec 15;11(6):e03027-20. doi: 10.1128/mBio.03027-20 (PMC7773995; doi:10.1128/mBio.03027-20)
Supplement: FIG S4 [file mBio.03027-20-sf004.pdf]

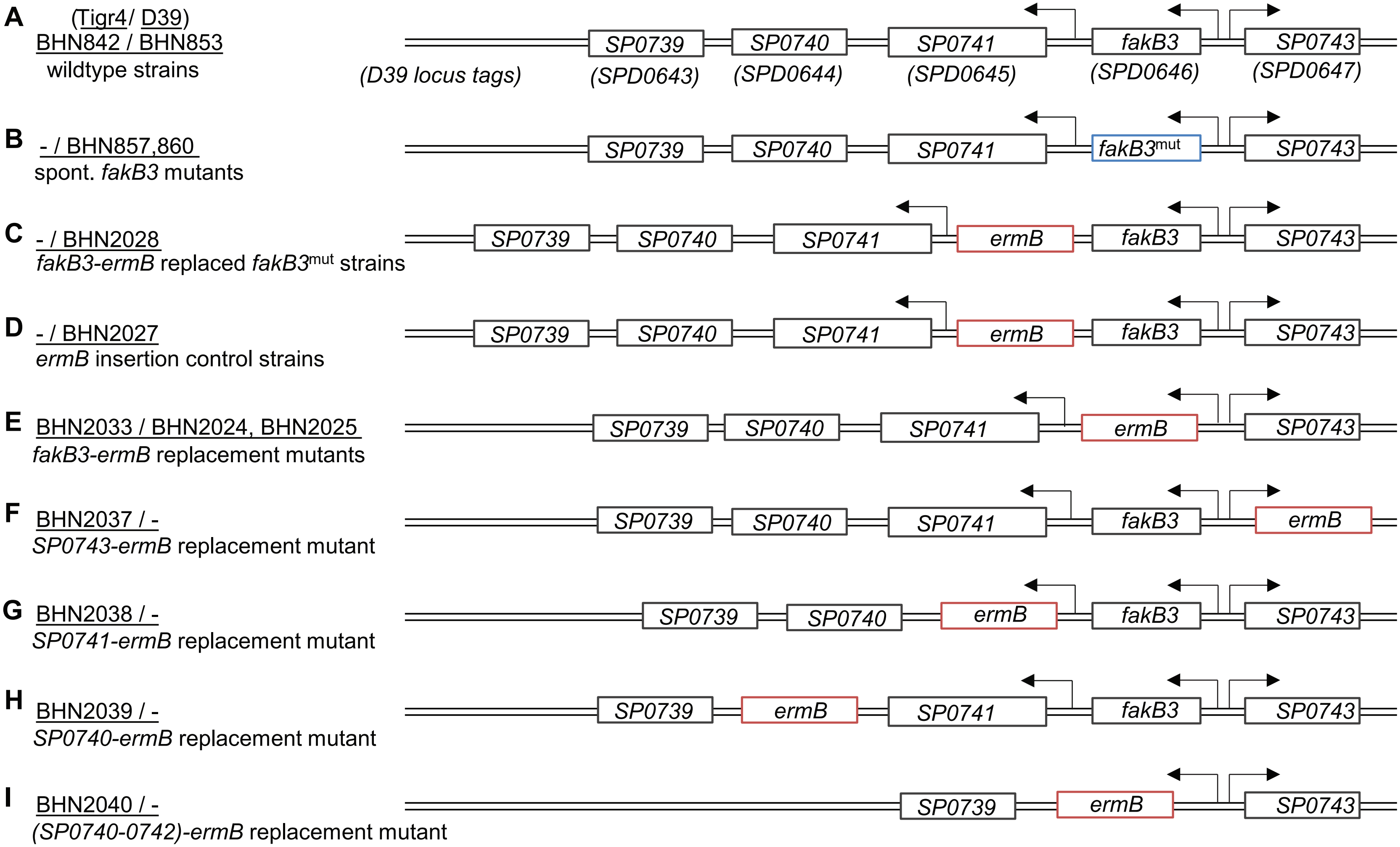

Figure S4. Overview of the *fakB3* locus and neighboring genes in the wildtype and mutant strains produced in the study.
(A) The gene segment covering Orfs *SP0739-43* (Tigr4) and *SPD0643-47* (D39) including *fakB3* and neighboring Orfs with location of promotors indicated with arrows. **(B)** Strains BHN857 and BHN860 (D39) contain spontaneous *fakB3* mutations selected after 2CCA-1 treatment. **(C)** Strain BHN2028 is a derivative of the spontaneous *fakB3* mutant strain BHN857 where the mutant *fakB3* allele was replaced with a wildtype *fakB3* allele. An *ermB* cassette was inserted between *fakB3* and *SP0741* Orfs to facilitate selection of transformants. **(D)** Control strain (BHN2027) in which an *ermB* cassette was inserted between *fakB3* and *SP0741* Orfs in the D39 (BHN853) background. **(E)** Mutants (BHN2033 (Tigr4) and BHN2024, BHN2025 (D39)) in which the *fakB3* Orf was replaced with an *ermB* cassette. Gene replacement mutants in which **(F)** Orf *SP0743*, **(G)** Orf *SP0741* and **(H)** Orf *SP0740* were individually replaced with an *ermB* cassette to generate strains BHN2037, BHN2038 and BHN2039, respectively. **(I)** Strain BHN2040 in which the gene segment including Orfs *SP0740-SP0742* was replaced with an *ermB* cassette.
