# Supplementary material for: The Bactericidal Fatty Acid Mimetic 2CCA-1 Selectively Targets Pneumococcal Extracellular Polyunsaturated Fatty Acid Metabolism
Source: mBio. 2020 Dec 15;11(6):e03027-20. doi: 10.1128/mBio.03027-20 (PMC7773995; doi:10.1128/mBio.03027-20)
Supplement: FIG S5 [file mBio.03027-20-sf005.pdf]

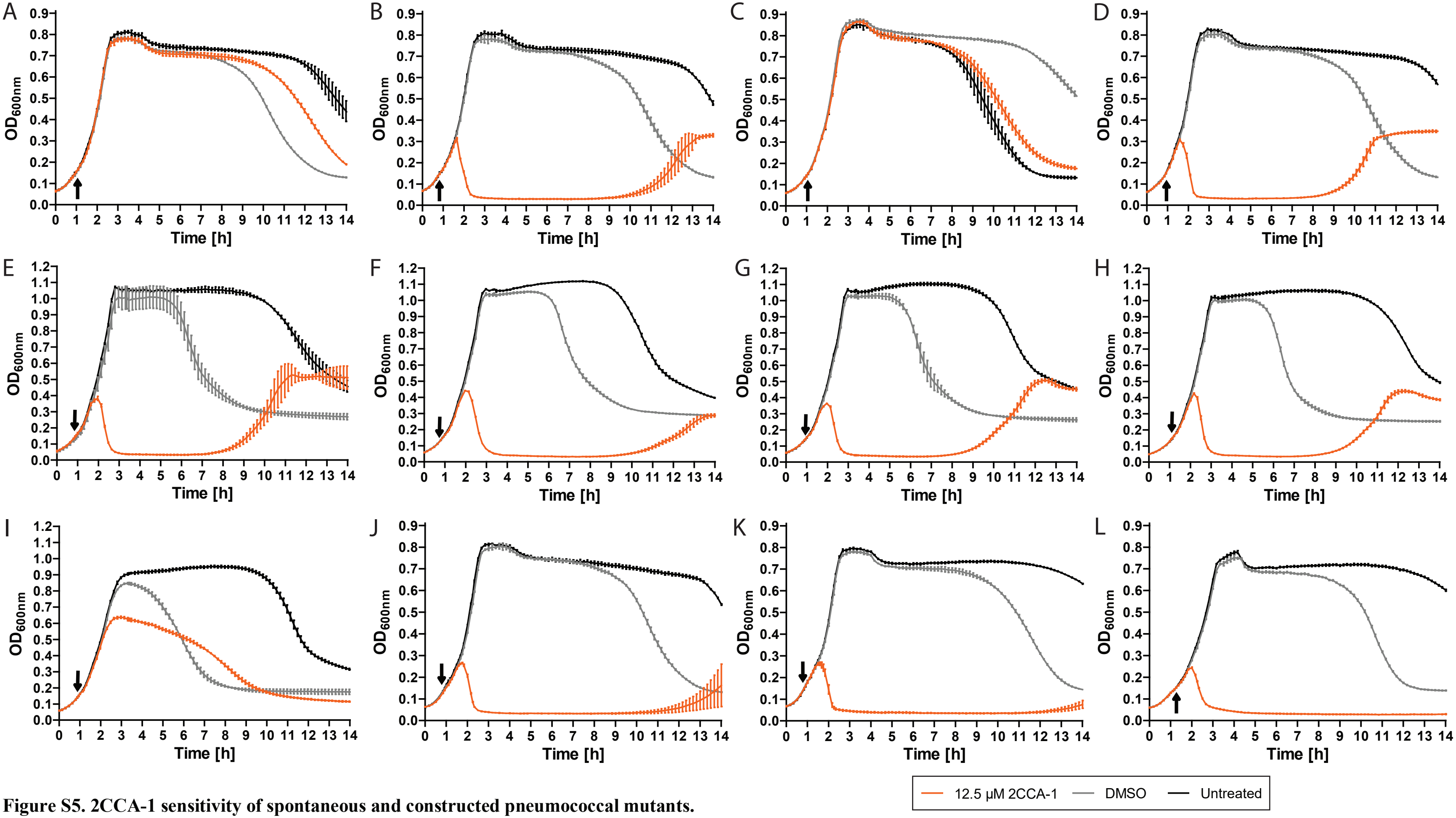


**Figure S5. 2CCA-1 sensitivity of spontaneous and constructed pneumococcal mutants.**
All strains were treated with 2CCA-1 (12.5 µM) and DMSO (1 % v/v) as compound solvent control. **(A)** The spontaneous 2CCA-1 resistant D39 derivative strain BHN857 containing a 97bp deletion in *fakB3* and **(B)** the strain with an introduced wildtype version of *fakB3* into the same locus (BHN2028). **(C)** The spontaneous 2CCA-1 resistant D39 derivative strain BHN859 with a non-synonymous point mutation in *fabT* and **(D)** the strain where the mutant allele was replaced with a wildtype allele in the *fabT* locus (BHN 2023). Control strain BHN2025 was not affected by 2CCA-1 and control strains BHN2022 and BHN2027 responded with lysis to 2CCA-1 treatment (data not shown). Corresponding strains in the Tigr4 background where the *fabT* mutant allele of the spontaneous 2CCA-1 resistant strain BHN848 was replaced wildtype allele (BHN2036) as well as the control strain BHN2035 responded with lysis to 2CCA-1 treatment whereas strains with a knockout in *fakB3* (BHN2033) and *fabT* (BHN2034) were resistant to 2CCA-1 (data not shown). Knockouts of genes affected by the 1639 bp (BHN870) and 4006 bp (BHN851) deletions in vicinity to *fakB3* with **(E)** the parental Tigr4 (BHN842), **(F)** T4Δ*SP0743::ermB* (BHN2037), **(G)** T4Δ*SP0741::ermB* (BHN2038), **(H)** T4Δ*SP0740::ermB* (BHN2039) and **(I)** T4Δ*SP0740-SP0742::ermB* (BHN2040). Knockouts of genes encoding pneumococcal fatty acid binding proteins **(J)** *fakB1* (BHN2030), **(K)** *fakB2* (BHN2031) and **(L)** the double knockout of those genes (BHN1351). Arrows indicate the timepoint of treatment administration. Avg +/- SD of triplicate treatment in one biological experiment are shown.
