# Supplementary material for: The Bactericidal Fatty Acid Mimetic 2CCA-1 Selectively Targets Pneumococcal Extracellular Polyunsaturated Fatty Acid Metabolism
Source: mBio. 2020 Dec 15;11(6):e03027-20. doi: 10.1128/mBio.03027-20 (PMC7773995; doi:10.1128/mBio.03027-20)
Supplement: FIG S6 [file mBio.03027-20-sf006.pdf]

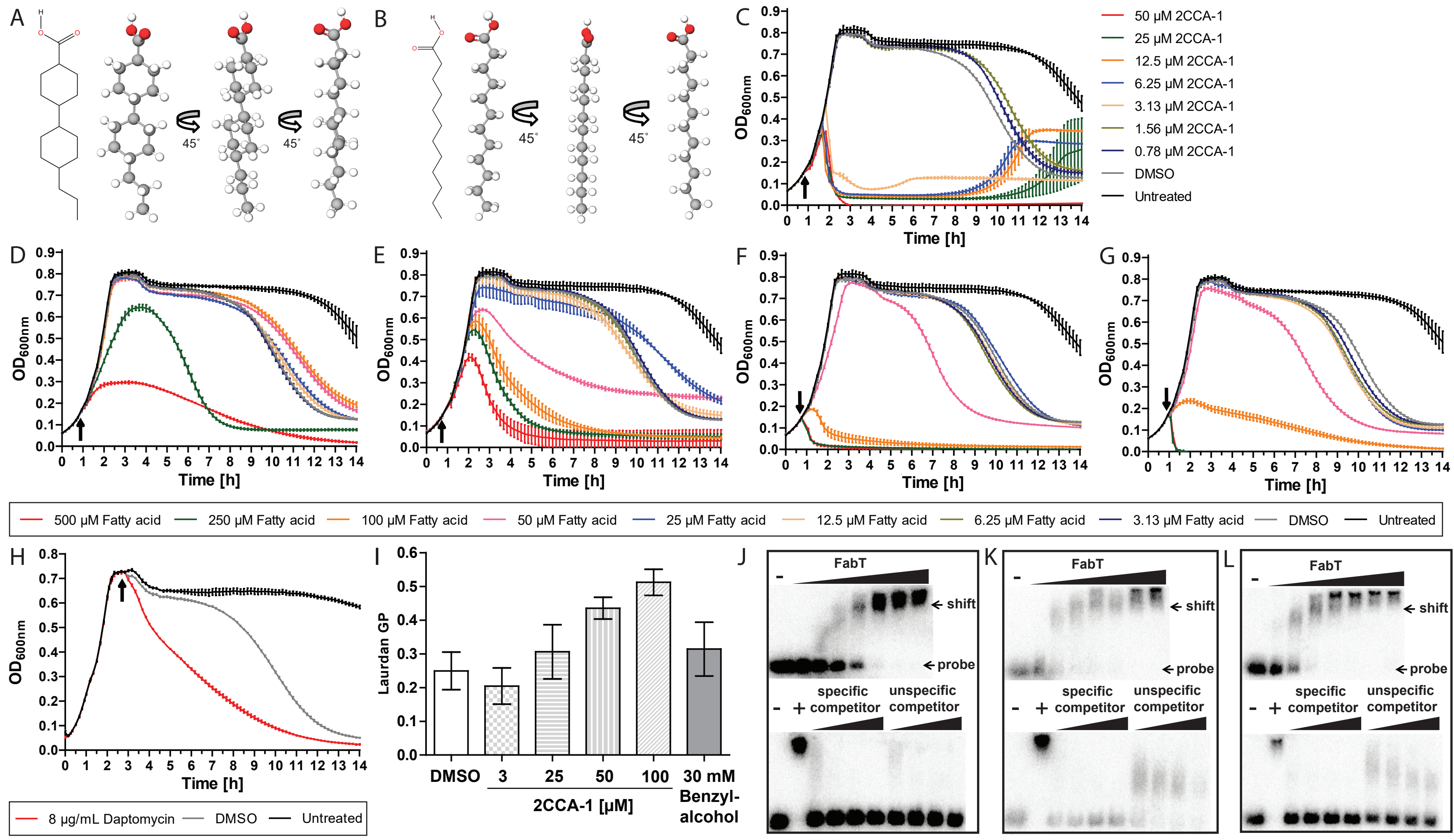

Figure S6. The fatty acid mimetic 2CCA-1.

(A) 2CCA-1 and **(B)** lauric acid (12:0) in three dimensional representations. **(C)** 2CCA-1, **(D)** lauric acid (12:0), **(E)** stearic acid (18:0), **(F)** oleic acid (18:1Δ9) and **(G)** linoleic acid (18:2Δ9, 12) added in a concentration titration to D39 grown in supplemented C+Y medium to assess the lysis inducing activity. **(H)** Treatment of D39 cultures in stationary phase with daptomycin (8 µg/mL) and DMSO (1 % v/v) as solvent control in supplemented C+Y medium with Ca^{2+}(50 µg/mL). Arrows indicate the timepoint of treatment administration. Avg +/- SD of triplicate treatment in one biological experiment are shown. **(I)** Determination of laurdan generalized polarization (GP) to record of changes in membrane fluidity upon treatment with 2CCA-1 (3 µM (1 x MlytC), 25 µM (8 x MlytC), 50 µM (16 x MlytC) and 100 µM (32 x MlytC)). Treatments with 1 % DMSO as solvent control and 30 mM benzyl alcohol as positive control for membrane fluidization were included. Avg +/- SD of at least biological triplicates (performed in at least technical duplicates) are shown **(J-L)** Electro mobility shift assay (EMSA) with **(J)** a 300 bp fragment including the *fakB3* promoter and 300 bp fragments of a sequence derived from the GAPDH orf in which FabT binding sites of either **(K)** the *fabT* promoter or **(L)** the *fabK* promoter were included. The radioactively end-labelled probe (0.4 nM) was incubated with no FabT (-) and increasing concentrations of FabT (2.5 nM, 5 nM, 7.5 nM, 10 nM, 15 nM, 20 nM and 25nM). The lower panels show competition conditions for FabT binding. Increasing concentrations (4 nM, 8 nM, 12 nM and 16 nM) of unlabeled probe as specific competitor and a 300bp GAPDH fragment with no FabT binding site as unspecific competitor were added to 25 nM FabT before the labelled probe (0.4 nM). The labelled probe alone (-) and the shift with 25 nM FabT (+) are shown for reference. Representative gels are shown. A band shift was observed when FabT was incubated with the radioactively end-labelled probe (**J-L**, upper panels) of the *fakB3* promoter and fragments containing the FabT binding sites within the *fabT* and *fabK* promoter. However, competition experiments (**J-L**, lower panels) showed that, in contrast to FabT binding to the *fabT* and *fabK* promoter, binding to the *fakB3* promoter was not specific.
